# Identification of a localized nonsense-mediated decay pathway at the endoplasmic reticulum

**DOI:** 10.1101/gad.338061.120

**Published:** 2020-08-01

**Authors:** Dasa Longman, Kathryn A. Jackson-Jones, Magdalena M. Maslon, Laura C. Murphy, Robert S. Young, Jack J. Stoddart, Nele Hug, Martin S. Taylor, Dimitrios K. Papadopoulos, Javier F. Cáceres

**Affiliations:** MRC Human Genetics Unit, Institute of Genetics and Molecular Medicine, University of Edinburgh, Edinburgh EH4 2XU, United Kingdom

**Keywords:** nonsense-mediated decay (NMD), RNA quality control, UPF1, NBAS, ER stress, UPR

## Abstract

Nonsense-mediated decay (NMD) is a translation-dependent RNA quality control mechanism that occurs in the cytoplasm. However, it is unknown how NMD regulates the stability of RNAs translated at the endoplasmic reticulum (ER). Here, we identify a localized NMD pathway dedicated to ER-translated mRNAs. We previously identified NBAS, a component of the Syntaxin 18 complex involved in Golgi-to-ER trafficking, as a novel NMD factor. Furthermore, we show that NBAS fulfills an independent function in NMD. This ER–NMD pathway requires the interaction of NBAS with the core NMD factor UPF1, which is partially localized at the ER in the proximity of the translocon. NBAS and UPF1 coregulate the stability of ER-associated transcripts, in particular those associated with the cellular stress response. We propose a model where NBAS recruits UPF1 to the membrane of the ER and activates an ER-dedicated NMD pathway, thus providing an ER-protective function by ensuring quality control of ER-translated mRNAs.

## Abstract (precis)

In this study, Longman et al. sought to understand how NMD regulates the stability of RNAs translated at the endoplasmic reticulum (ER), and identified a localized NMD pathway dedicated to ER-translated mRNAs. They show that NBAS, a component of the Syntaxin 18 complex involved in Golgi-to-ER trafficking, fulfills an independent function in NMD, and propose a model where NBAS recruits UPF1 to the membrane of the ER and activates an ER-dedicated NMD pathway, thus providing an ER-protective function by ensuring quality control of ER-translated mRNAs.

The nonsense-mediated decay (NMD) pathway is a highly conserved surveillance mechanism that targets mRNAs harboring premature termination codons (PTCs) for degradation. In doing so, it prevents the accumulation of truncated proteins and modulates the phenotypic outcome of genetic disorders that arise due to the presence of PTCs (Bhuvanagiri et al. 2010). Importantly, NMD also controls the stability of a large number of endogenous RNAs and fine-tunes many physiological processes (for review, see Karousis and Mühlemann 2019; Kurosaki et al. 2019).

Core NMD factors were initially identified in genetic screens in *C. elegans* and in *S. cerevisiae* and were later shown to be involved in NMD in other species, including *Arabidopsis*, *Drosophila*, and mammals. Human orthologs include SMG1, UPF1, UPF2, UPF3, SMG5, SMG6, and SMG7. Additional NMD factors have been identified using a variety of different experimental approaches, including interactome studies, RNAi screens in nematodes, and CRISPR screens in mammalian cells (Hug et al. 2016; Alexandrov et al. 2017; Baird et al. 2018). Mechanistically, NMD is tightly linked to mRNA translation and is initiated by the recognition of a PTC by the surveillance (SURF) complex within which the RNA helicase UPF1 and its associated kinase, SMG1, bind to the ribosomal release factors eRF1 and eRF3 (Karousis and Mühlemann 2019). Subsequently, components of the SURF complex interact with the core NMD factors UPF2 and UPF3B, and with an exon junction complex (EJC) located downstream from the PTC, to form the decay-inducing complex (DECID) that triggers UPF1 phosphorylation by SMG1, and dissociation of eRF1 and eRF3 (Kashima et al. 2006; Singh et al. 2007). This leads to the recruitment of mRNA degradation factors that trigger RNA decay. Substrate selection for NMD occurs not only in an EJC-dependent manner, but alternatively via an EJC-independent mechanism, which targets transcripts harboring very long 3′ UTRs (Metze et al. 2013; Kurosaki et al. 2019).

Using RNAi screens in *C. elegans*, we previously identified several novel NMD factors that were also essential for viability, suggesting that they fulfill additional functions in nematodes, where this pathway is not essential (Longman et al. 2007; Casadio et al. 2015). One of these novel NMD factors is encoded by *smgl-1* that corresponds to the human gene *NBAS* (neuroblastoma amplified sequence, also known as *NAG*, for neuroblastoma amplified gene). *NBAS* was first identified as a gene that is coamplified with the *N-myc* gene in human neuroblastomas; however, no clear role in the disease has been reported (Wimmer et al. 1999; Scott et al. 2003). We previously showed that NBAS acts in concert with UPF1 to coregulate a large number of transcripts not only in nematodes but also in zebrafish and human cells (Anastasaki et al. 2011; Longman et al. 2013). *NBAS* encodes a peripheral ER membrane protein that is a component of the Syntaxin 18 complex, which functions in Golgi-to-ER retrograde transport (Aoki et al. 2009). A series of loss-of-function mutations in *NBAS* have been found in several human conditions, including biallelic mutations in patients with a multisystem disease involving liver, eye, immune system, connective tissue, and bone (Haack et al. 2015; Segarra et al. 2015). Compound heterozygous variants in *NBAS* were also identified as a cause of atypical osteogenesis imperfecta (Balasubramanian et al. 2017) and in a short stature with optic atrophy and Pelger-Huët anomaly (SOPH) syndrome (Maksimova et al. 2010). Currently, it remains unclear whether the phenotypes observed in patients with mutations in *NBAS* are due to a compromised NMD response, defects in Golgi-to-ER retrograde transport, or a combination of both.

Despite initial controversy concerning the intracellular location of NMD in mammalian cells, it has been conclusively demonstrated that decay of a PTC-containing β-globin NMD reporter occurs in the cytoplasm (Trcek et al. 2013). The ER is a major site of localized protein synthesis, with approximately a third of all mRNAs being translated there, in particular, those encoding proteins entering the secretory pathway. It has become increasingly evident that ER and cytosol constitute different environment for protein translation and posttranscriptional gene regulation (Reid and Nicchitta 2015). Current efforts have focused on the mechanism and regulation of cytoplasmic NMD; however, it is largely unknown how this mechanism operates on mRNAs that are translated at the ER, which, due to their intrinsic localized translation, will not have sufficient exposure to cytoplasmic NMD. There is a precedent for a localized NMD response in neurons, where NMD regulates the expression of both dendritic and axonal mRNAs upon their activation of localized mRNA translation (Giorgi et al. 2007; Colak et al. 2013). Both NBAS and a second novel NMD factor identified in our RNAi screens, SEC13 (nuclear pore and COPII coat complex component), localize to the membrane of the ER, raising the possibility that they could be involved in an ER-localized NMD pathway (Longman et al. 2007; Casadio et al. 2015).

Here, we present evidence that reveals a central role for NBAS, acting together with UPF1, in an NMD response that is associated with the ER. We show that NBAS has dual roles in NMD and Golgi-to-ER retrograde transport; but importantly, these functions act independent of each other. We demonstrate that NBAS recruits the core NMD factor UPF1 to the membrane of the ER and promotes the degradation of NMD substrates that are translated at the ER.

## Results

### A dual role of NBAS in Golgi-to-ER transport and NMD

**Figure 1.**
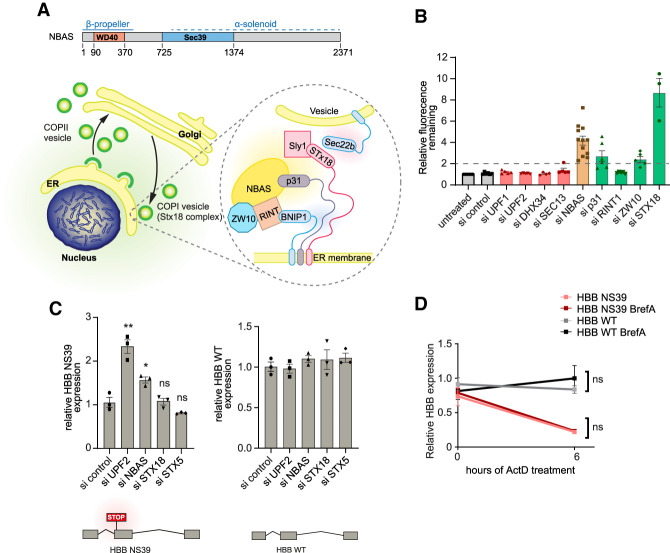
NBAS is an ER-localized protein with dual, independent, functions in NMD and ER secretion. (*A*) Cartoon depicting the functional domains of NBAS and its intracellular localization at the endoplasmic reticulum (ER), as part of the Syntaxin 18 complex. (*B*) NMD activity is not required for constitutive secretion. Depletion of NMD factors, with the exception of NBAS, does not affect the ability of HeLa C1 cells to secrete a GFP-based reporter construct. GFP accumulation was measured by flow cytometry and a secretion defect was defined as a twofold accumulation of GFP reporter (after the addition of 1 µm D/D solubilizer [ligand] compared with untreated HeLa C1 cells [dashed line]). Each point represents one biological replica, bars indicate mean with SEM. (*C*) NMD activity is not affected by depletion of secretion factors, STX18 and STX5. In contrast, depletion of UPF2 and NBAS led to an up-regulation of the HBB NS39 NMD-sensitive reporter. HeLa cells stably expressing the HBB NS39 or wild-type (WT) reporters were depleted of the indicated factors and the steady state level of the reporter mRNA was measured by qRT-PCR and normalized to POLR2J expression. Each point represents one biological replica, bars indicate mean with SEM. Significance was determined by two-tailed unpaired *t*-test. (**) *P* < 0.005; (*) *P* < 0.05; (ns) not significant. (*D*) NMD activity is not affected by blocking constitutive secretion. HeLa cells expressing HBB WT or HBB NS39 reporters were treated with actinomycin D to block transcription and Brefeldin A to block constitutive secretion. HBB expression was measured as described in *C*. Each point represents the mean and SEM of three biological replicas. The significance of Brefeldin A treatment was determined by two-tailed unpaired *t*-test. (ns) Not significant. See Supplemental Figure S1C for the effect of Brefeldin A on secretion.

*NBAS* encodes a 2371 amino acid protein containing WD40 repeats and a SEC39 domain, present in proteins involved in the secretory pathway (Fig. 1A). Together with RINT1 and ZW10, NBAS forms part of the evolutionarily conserved NRZ complex, which functions as a tethering complex for retrograde trafficking of COPI vesicles from the Golgi to the ER and is part of the larger Syntaxin 18 complex (Aoki et al. 2009; Çivril et al. 2010).

To dissect whether NBAS had separate roles in NMD and Golgi-to-ER transport, or whether these processes influence each other, we first tested whether abrogating the NMD pathway influences ER trafficking. We used a modified flow cytometry-based assay that relies on the expression of an eGFP fluorescent reporter for measuring constitutive secretion. This assay is based on the property of mutant FKBP (FK506-binding protein) (F36M) to form large aggregates that, when expressed in the ER, cannot be secreted. However, upon incubation with an FKBP (F36M) ligand, D/D solubilizer, these aggregates are solubilized, leading to efficient secretion (Gordon et al. 2010). We used this reporter in combination with siRNA-mediated knockdown of individual NMD factors or of components of the Syntaxin 18 complex (Fig. 1B; Supplemental Fig. S1A). Fluorescence of clonal HeLa C1 cells stably expressing the eGFP reporter decreased upon addition of the ligand, D/D solubilizer, to the level of control cells, as was observed previously (Supplemental Fig. S1A, left panel; Gordon et al. 2010). As expected, siRNA-mediated knockdown of components of the Syntaxin 18 complex, including STX18, p31, and NBAS, resulted in an increase of the remaining fluorescence due to an interference with the secretion process (Fig. 1B). We observed some effect upon depletion of ZW10, but almost no effect with the knockdown of RINT1, perhaps reflecting a differential contribution of these components to the secretion process. Importantly, we observed that a strong reduction in the levels of individual NMD factors did not interfere with secretion. This was the case for UPF1 and UPF2, but also for the RNA helicase DHX34, and for SEC13, another NMD factor that localizes to the ER (Fig. 1B; Supplemental Fig. S1A). From this experiment, we conclude that NMD activity is not required for ER secretion.

Next, we investigated whether interfering with constitutive secretion had any effect on the NMD pathway. For this, HeLa cells stably expressing a well-characterized β-globin NMD reporter harboring a nonsense mutation at position 39 (HBB NS39) (Trecartin et al. 1981), or its wild-type (WT) HBB counterpart, were depleted of NMD factors (UPF2 or NBAS) or of secretion factors (STX18 or STX5) using specific siRNAs (Supplemental Fig. S1D). As expected, the level of HBB WT mRNA remained unchanged upon UPF1, NBAS, STX18, or STX5 depletion (Fig. 1C, right panel). In contrast, depletion of UPF2 or of NBAS resulted in an increased level of the HBB NS39 NMD reporter mRNA, compared with mock-depleted cells (Fig. 1C, left panel). Importantly, depletion of either STX18 or STX5 did not affect the level of β-globin NMD reporter mRNA (Fig. 1C), indicating that normal ER secretion is not required for NMD. These results were confirmed using a fluorescent NMD reporter (NMD^+^) that quantifies NMD activity at the single cell level. Cells carrying the NMD^+^ reporter were identified by the constitutive expression of red fluorescence, whereas NMD activity was determined by the mean green fluorescence, which is subject to NMD, in all red cells (Pereverzev et al. 2015). Here again, whereas knockdown of UPF2 and NBAS resulted in increased levels of the NMD reporter (measured by increased green fluorescence), depletion of STX18 or STX5 had no effect (Supplemental Fig. S1B). We extended these observations to show that NMD activity is not affected by blocking constitutive secretion with the use of Brefeldin A (Supplemental Fig. S1C; Aridor et al. 1995). HeLa cells stably expressing either HBB WT or HBB NS39 were treated with actinomycin D to block transcription in the presence or absence of Brefeldin A. We observed that the stability of the HBB NS39 NMD reporter mRNA was not increased by blocking ER secretion (Fig. 1D). Altogether, these experiments show that NMD activity and ER secretion are not functionally linked, strongly suggesting that NBAS has two independent roles in Golgi-ER retrograde transport and in NMD.

### NBAS regulates a subset of NMD targets specifically translated at the ER

**Figure 2.**
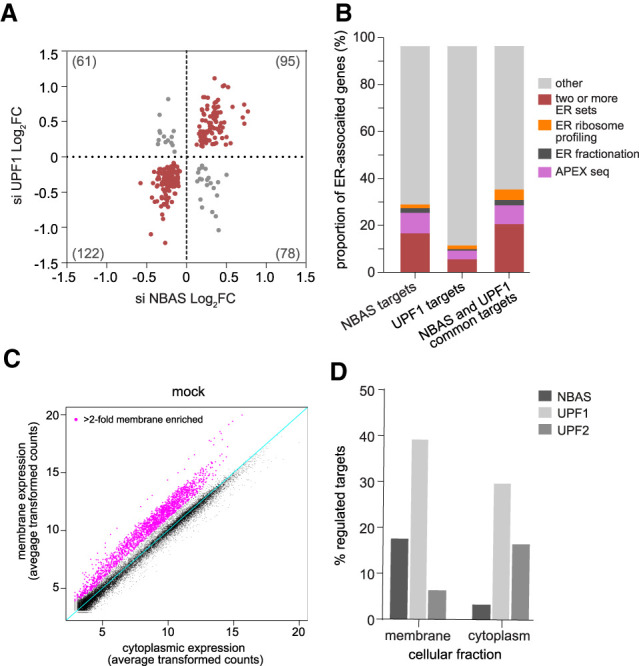
NBAS-regulated targets are enriched for ER-localized transcripts. (*A*) Scatter plot of differentially expressed transcripts shows a significant positive correlation between NBAS and UPF1-regulated targets (Pearson's correlation *r* = 0.67; *P* < 0.0001). Numbers in brackets indicate the number of regulated transcripts in each quadrant. (*B*) NBAS-regulated targets are enriched for experimentally identified ER-localized transcripts. Bar charts show the proportion of targets that are ER-localized by APEX-seq, ER fractionation, and ER proximity-specific ribosome profiling. The ER enrichment of NBAS targets is significantly higher than that of UPF1 targets (*P* < 0.0001, exact binomial test), whereas the difference between NBAS targets and NBAS-UPF1 common targets is not statistically significant (*P* = 0.1867, exact binomial test). (*C*) RNA-seq of samples from subcellular fractions (membrane and cytoplasm) allows identification of membrane-associated genes. Scatter plot shows average transformed counts of each gene in membrane and cytoplasmic fractions in mock-depleted cells. (*D*) NBAS preferentially regulates membrane-associated targets. Plot shows percentage of “membrane-associated” genes in the membrane fraction and of “nonmembrane” genes in the cytoplasm that are regulated (*P* < 0.05) by each NMD factor. NBAS regulates over fivefold higher percentage of membrane-associated genes than nonmembrane genes (*P* < 0.001, exact binomial test). In contrast, UPF1 regulates a similar percentage of both membrane-associated and nonmembrane genes (1.3-fold higher percentage of membrane-associated genes, *P* < 0.001, exact binomial test), and UPF2 regulates 2.5-fold higher percentage of nonmembrane genes than membrane genes (*P* < 0.001, exact binomial test).

Previously, we conducted RNA profiling experiments that revealed a large proportion of NBAS mRNA targets are coregulated by the core NMD factor UPF1, with a significant enrichment for genes involved in the cellular stress response (Longman et al. 2013). Here, we extended this analysis by RNA sequencing to profile changes in mRNA abundance upon depletion of NBAS or UPF1. Both NBAS and UPF1 were the most significantly down-regulated genes in the relevant samples (fold change −1.85, *P* < 3.51 × 10^−13^, and fold change −3.28, *P* < 1.14 × 10^−148^, respectively) (Supplemental Table S1). Depletion of UPF1 significantly affected the mRNAs of 4756 genes, with 2411 genes displaying an increased mRNA expression upon UPF1 knockdown. Depletion of NBAS increased the mRNA levels of 209 genes (Supplemental Table S1), consistent with the view that NBAS regulates a subset of the UPF1 targets. We observed a robust coregulation of mRNAs when UPF1 or NBAS was depleted (Pearson's correlation *r* = 0.67, *P* < 0.0001) (Fig. 2A), indicating that UPF1 and NBAS function in a common pathway. Gene ontology (GO) analysis of the mRNAs regulated by both NBAS and UPF1 revealed a strong enrichment for the secretome, which is preferentially translated at the ER translocon (Supplemental Table S2). Interestingly, we found a significant increase in fold change in genes associated with the ER unfolded protein response (UPR) (GO: 0006986) when either NBAS or UPF1 was depleted (*P* < 0.05 and *P* < 0.001, respectively; Wilcoxon rank sum test), showing that the ER stress response is perturbed when the NMD response at the ER is not functional (Supplemental Fig. S2). This supports our hypothesis that an ER-specific NMD pathway is essential for cellular homeostasis.

To further characterize genes regulated by NBAS and UPF1, we intersected targets regulated by either NBAS or UPF1 and also common targets, with three experimental data sets identifying genes localized and/or translated at the ER. APEX-seq, a method for RNA sequencing based on direct proximity labeling of RNA led to the identification of 1077 mRNAs localized at the ER (Fazal et al. 2019), cell fractionation followed by ribosome footprinting identified 486 mRNAs (Reid and Nicchitta 2012), and proximity-specific ribosome profiling, based on ER membrane proximity labeling of ribosomes, identified 686 mRNAs translated at the ER (Jan et al. 2014). We found that NBAS targets are strongly enriched for experimentally validated ER genes (31.6%), as compared with 14.4% of UPF1 targets. Furthermore, 18.2% of NBAS targets were found in two or more of the data sets, as compared with only 6.3% of UPF1 targets. Considering NBAS and UPF1 common targets, 37.9% of targets were found in at least one data set, while 23.2% were found in two or more data sets (Fig. 2B). NBAS-regulated targets were strongly enriched for experimentally identified ER-localized genes (OR = 6.07, *P* < 0.001, Fisher's exact test), whereas UPF1 targets were only modestly enriched (OR = 2.44, *P* < 0.001, Fisher's exact test). Compared with UPF1 targets, we found that those targets that were also regulated by NBAS were 2.6-fold more likely than expected by chance to be found at the ER (*P* < 0.001, exact binomial test), confirming that NBAS and UPF1 together regulate NMD targets specifically at the ER.

We next examined how NBAS depletion affected transcripts specifically at the ER compared with the cytoplasm, and how this differs from depletion of core NMD factors UPF1 and UPF2. We performed RNA sequencing on cytoplasmic and membrane fractions of HeLa cells depleted for each factor or mock-depleted control cells, following subcellular fractionation. By comparing expression of genes in each fraction in control samples we defined a group of “membrane-associated” genes, as those that in the control sample showed a twofold higher expression in the membrane fraction (magenta points), whereas all other expressed genes were deemed “nonmembrane” genes (Fig. 2C). We assessed the validity of cell fractionation by identifying genes in the previously described experimentally validated ER data sets in our data. As expected, we observed that the majority of genes present in two or more ER data sets were found in the membrane-associated set (OR = 66.94, *P* < 0.0001, Fisher's exact test) (Supplemental Fig. S3A). Next, we analyzed changes in gene expression of “membrane-associated” genes in the membrane fraction, and of “nonmembrane” genes in the cytoplasmic fraction, upon depletion of individual NMD factors (Fig. 2D; Supplemental Fig. S3B,C; Supplemental Table S3). Knockdown of UPF1 led to a robust increase in the abundance of mRNAs in both membrane and cytoplasmic fractions, whereas depletion of NBAS led to an up-regulation of a higher percentage of membrane-associated genes than of nonmembrane genes (fivefold increase, *P* < 0.001, exact binomial test). In contrast, depletion of UPF2 led to preferential up-regulation of “nonmembrane” genes in the cytoplasm (>2.5-fold, *P* < 0.001, exact binomial test). Altogether, these results suggest that NBAS is a crucial component of an ER–NMD pathway that targets for degradation mRNAs that are translated at the ER, constituting a novel localized NMD response.

### Site of NMD for transcripts translated at the ER

**Figure 3.**
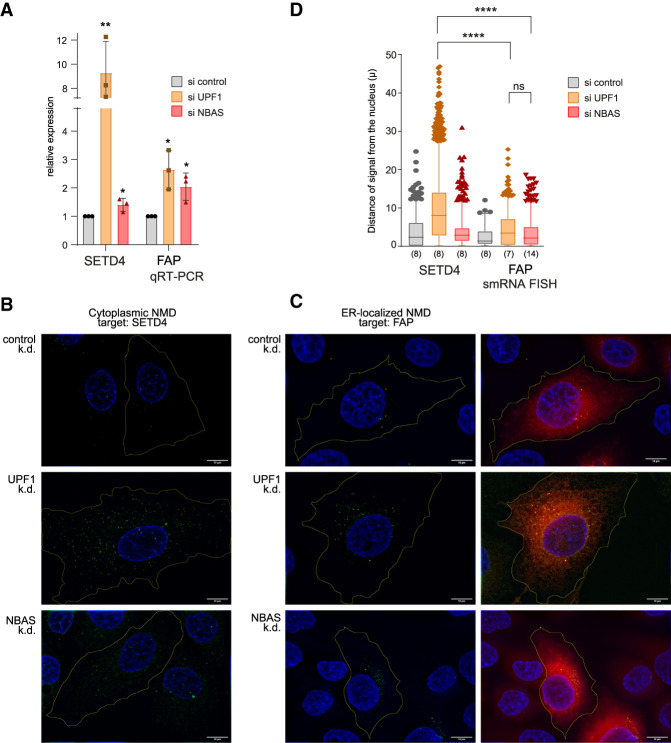
ER-localized NMD targets are degraded at the ER. (*A*) Relative changes in SETD4 and FAP expression upon UPF1 or NBAS depletion were measured by qRT-PCR and normalized to POLR2J expression. Each point represents one biological replica, bars indicate mean with SEM. Significance was determined by two-tailed unpaired *t*-test. (**) *P* < 0.005; (*) *P* < 0.05. (*B,C*) Single-molecule (sm) RNA FISH was used to visualize the site of accumulation of a cytoplasmic NMD target SETD4 (*B*), or an ER-localized NMD target FAP (*C*), within the cell when NMD was abrogated by either UPF1 or NBAS knockdown. Individual RNA molecules were visualized by a set of transcript-specific Quasar 570-labeled Stellaris probes and pseudocolored green. The cell outline was visualized by staining with MemBright 640-nm probe or anti-Sec61B antibody and is indicated by the yellow line, drawn using Fiji (for more details, see the Materials and Methods). (*Right* column) The ER compartment was visualized by mEmeral-ER-3 cotransfected plasmid and is pseudocolored red. Scale bars, 10 µm. (*D*) Distribution of FISH signal within imaged cells is represented by the distance of signal from the nucleus in each condition. Graph shows distances as 5–95 percentile box plots, numbers in brackets indicate the number of analyzed cells. Data were acquired in three independent experiments. Statistical significance was determined by one-way ANOVA test. (****) *P*_adj < 0.0001; (ns) not significant.

Single-molecule RNA fluorescent in situ hybridization (smRNA FISH) was used previously to localize the NMD response of a β-globin NMD reporter to the cytoplasm (Trcek et al. 2013). Here, we used smRNA FISH to spatially map the location of mRNA degradation of mRNAs that are translated at the ER and that we also showed to be up-regulated upon UPF1 and/or NBAS depletion (Longman et al. 2013). We used a set of fluorescent probes that label the full-length of two endogenous mRNAs targeted by NMD, which are translated either in the cytoplasm or at the ER. We selected *SETD4* (SET Domain Containing 4) mRNA, which encodes a lysine methyltransferase and is translated in the cytoplasm (Faria et al. 2013). As an example of an mRNA that is translated at the ER, we selected *FAP* mRNA (also known as seprase), which encodes fibroblast activation protein α, a 170-kDa membrane-bound gelatinase (Goldstein et al. 1997). First, we showed that levels of *SETD4* mRNA were robustly increased upon knockdown of UPF1, whereas NBAS depletion had only a marginal stabilizing effect (Fig. 3A). In contrast, the ER-localized *FAP* mRNA was comparably up-regulated upon knockdown of UPF1 and of NBAS (Fig. 3A).

RNA FISH of *SETD4* mRNA in UPF1-depleted cells revealed a strong increase of uniformly distributed fluorescent signal throughout the cell, consistent with NMD taking place in the cytoplasm. As expected, depletion of NBAS led only to a slight up-regulation of *SETD4* mRNA (Fig. 3B). Importantly, RNA FISH of the ER-translated *FAP* mRNA in both UPF1 and NBAS-depleted cells revealed a strong increase of the fluorescent signal that clustered to the perinuclear region of the cell (Fig. 3C, green signal). The RNA FISH fluorescence overlapped with ER staining (Fig. 3C, right panels), thus spatially mapping the mRNA degradation of an NBAS NMD target to the ER. This is consistent with an NBAS-dependent NMD-mediated degradation occurring at the ER. In order to represent the differences in the distribution of the smRNA FISH signal within cells, we plotted the distance of the fluorescent FISH signal from the edge of the nucleus (defined by DAPI staining), for each experimental condition. This demonstrated that UPF1 depletion leads to the accumulation of *SETD4* mRNA that is distributed widely within the cell, which is significantly different from the distribution pattern of the ER-associated *FAP* mRNA signal that clusters close to the nuclear periphery upon depletion of both NBAS and UPF1 (Fig. 3D). Finally, using smRNA FISH signal counts we confirmed the increase of *SETD4* and *FAP* transcripts in response to UPF1 and NBAS depletion (Supplemental Fig. S4). Altogether, these results provide strong evidence that NBAS has a role in NMD regulation of mRNAs translated at the ER.

### UPF1 is present at the ER

The involvement of the ER-associated factor NBAS in the NMD response, together with its coregulation of RNA targets with UPF1, led us to probe whether UPF1 localizes to the ER and interacts with NBAS. It has been previously reported that UPF1 localizes to the cytoplasm both in yeast (Atkin et al. 1995) and human cells (Applequist et al. 1997; Serin et al. 2001). However, there is evidence that UPF1 is also localized at the ER. First, UPF1 was found associated with cytoplasmic, but also with ER-bound polysomes (Jagannathan et al. 2014a). Moreover, a large-scale protein–protein interactome revealed that UPF1 interacts with components of the Syntaxin 18 complex, ZW10, p31 and STX18, where NBAS also resides (Aoki et al. 2009; Brannan et al. 2016). First, we tagged endogenous NBAS with an eGFP/ 3xFlag tag at its N terminus and following anti-GFP immunoprecipitation, we revealed the interacting proteins using mass spectrometry. This resulted in the identification of several components of the Syntaxin 18 complex as well as of SEC61A1, a component of the trimeric SEC61 complex at the ER translocon that interact with NBAS independently of the presence of RNA (Supplemental Fig. S5A; Supplemental Table S4; Hartmann et al. 1994). We did not observe a robust enrichment of NMD factors despite having demonstrated these interactions by other experimental approaches (see Fig. 5, below; Supplemental Figs. S6, S7). We hypothesize that this is due to the more transient nature of interactions between NBAS and NMD factors compared with components of the Syntaxin 18 complex.

**Figure 4.**
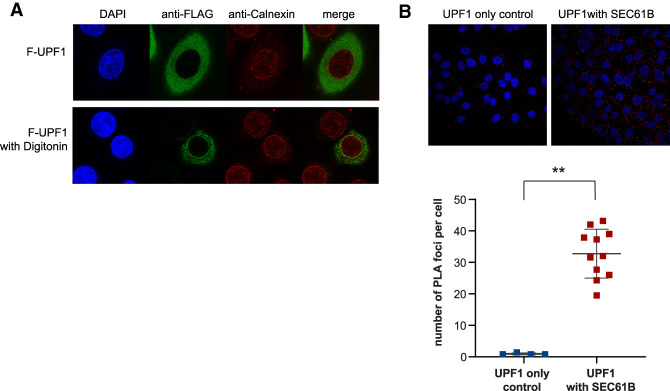
UPF1 localizes at the ER. (*A*) Immunofluorescence of HeLa cells transiently expressing Flag-tagged UPF1 (F-UPF1, in green) together with the ER marker Calnexin (in red). (*Top*) Cell nuclei were visualized by DAPI staining. UPF1 predominantly shows a diffused cytoplasmic localization. (*Bottom*) Partial cell permeabilization with digitonin revealed that a fraction of UPF1 is anchored at the ER membrane and colocalizes with calnexin. (*B*) UPF1 is localized in the close proximity of the SEC61B translocon component at the ER. Proximity ligation assay (PLA) using antibodies against endogenous UPF1 and SEC61B proteins generated a discrete signal (red spots), indicating that the proteins are <40 nm apart. The graph shows the quantification of PLA signal. Each point represents mean PLA count per cell in one captured frame. Significance was determined by two-tailed Mann-Whitney test. (**) *P*<0.005. Data showing the colocalization of NBAS and UPF2 with SEC61B are shown in Supplemental Figure S5.

Immunofluorescence analysis of HeLa cells transiently transfected with Flag-UPF1 showed that UPF1 localizes to the cytoplasm, as previously suggested. However, upon incubation of HeLa cells with digitonin, which permeabilizes the plasma membrane and consequently releases cytosolic components that are not anchored to cellular membranes, we observed a population of UPF1 that was resistant to digitonin treatment and colocalized with the ER marker calnexin, indicating ER membrane association (Fig. 4A). We next used the proximity ligation assay (PLA) to probe for interactions of UPF1 and NBAS, with SEC61B, a component of the SEC61 channel-forming translocon complex that is a central component of the protein translocation apparatus at the ER membrane. PLA has been extensively used to detect interactions of many cellular proteins, including the core NMD factors UPF1 and UPF2 (Tatsuno et al. 2016). We detected a robust PLA interaction between UPF1 and SEC61B in HeLa cells, indicating that UPF1 localizes at the site of mRNA translation at the ER (Fig. 4B). PLA analysis of the endogenously NBAS-tagged cell line revealed that NBAS is also colocalized with SEC61B (Supplemental Fig. S5B).

Altogether, these results show that both UPF1 and NBAS are present at the translocon, bringing them into close proximity with mRNAs being translated at the ER. Even though UPF1 is the core factor essential for NMD function, it is likely that other NMD factors will, at times, also partially localize to the ER. Previously, immunofluorescence was used to colocalize SMG6 and UPF3B with GRP78, a member of the heat shock protein 70 (HSP70) family that is present in the lumen of the ER (Sakaki et al. 2012). More recently, a large human interactome study identified UPF3B, in complex with UPF2 and UPF1, to be associated with SEC61A1 (Hein et al. 2015). To investigate whether other components of the NMD pathway with previously established cytoplasmic localization are also present at the ER, we probed for the colocalization of UPF2 with SEC61B. We observed a modest PLA signal indicating that a fraction of UPF2 is also associated with the translocon at the ER in HeLa cells (Supplemental Fig. S5C). This modest colocalization of UPF2 with the ER is consistent with our previous observation that UPF2 plays only a minor role in the regulation of ER-translated mRNAs (Fig. 2D).

### NBAS interacts with UPF1 and preferentially associates with the SURF complex

The presence of NBAS and UPF1 in proximity with the ER translocon, together with the coregulation of RNA targets by NBAS and UPF1 as part of the ER–NMD response strongly suggested an interaction of these two NMD factors. To investigate this we first tested for the copurification of NBAS with UPF1 by coimmunoprecipitation in HeLa cells, in the presence or absence of RNases. We found that Flag-tagged UPF1 coimmunoprecipitated with cotransfected T7-NBAS (Supplemental Fig. S6A) and we also detected the interaction of endogenous UPF1 with Flag-tagged NBAS, even in the absence of RNA (Supplemental Fig. S6B). Since UPF1 phosphorylation is a later step in NMD activation it can be used as a diagnostic tool to infer the timing of recruitment of a particular protein to the NMD complex. We used two UPF1 mutants that resemble the hypophosphorylated state of UPF1 when present in the early surveillance (SURF) complex (C126S), or the hyperphosphorylated UPF1 present in the late decay-inducing (DECID) complex (K498A) (Weng et al. 1996; Kashima et al. 2006). As observed previously, the C126S mutation blocks UPF1 interaction with UPF2, yet it binds to T7-tagged NBAS, whereas the K498A UPF1 mutant displayed no NBAS binding (Supplemental Fig. S6C). This strongly suggests that NBAS is preferentially associated with the initial surveillance SURF complex, where UPF1 is hypophosphorylated. Interestingly, we observed previously similar results for the RNA helicase DHX34 (Hug and Cáceres 2014), suggesting that most of the regulatory steps of the NMD pathway occur in the early stages of the NMD response. The observed biochemical interaction of NBAS with UPF1 was not RNA-dependent (Supplemental Fig. S6A,B). Nevertheless, we wanted to investigate the possibility that NBAS directly recruits NMD targets for degradation. We tested whether NBAS directly binds to mRNA using an mRNA capture assay, which relies on in-situ UV cross-linking, followed by affinity selection of mRNPs by oligo-dT cellulose (Piñol-Roma and Dreyfuss 1992; Sanford et al. 2005). Affinity selection of mRNPs by oligo-dT cellulose showed that NBAS binds to mRNA (Supplemental Fig. S6D), opening the possibility that RNA-binding by NBAS contributes to selection of NMD targets at the ER.

Next, we probed for the interaction of endogenous NBAS with other core NMD factors. We detected an interaction of transiently expressed Flag-tagged SMG5, SMG6, SMG7, and UPF1 with endogenous NBAS, even in the absence of RNA (Supplemental Fig. S7A). Additionally, we observed that endogenous NBAS can also be copurified with full-length tagged RNA helicase DHX34, but not with a truncated DHX34 version lacking an essential OB-like domain (Supplemental Fig. S7B), which is required for helicase function (Melero et al. 2016).

**Figure 5.**
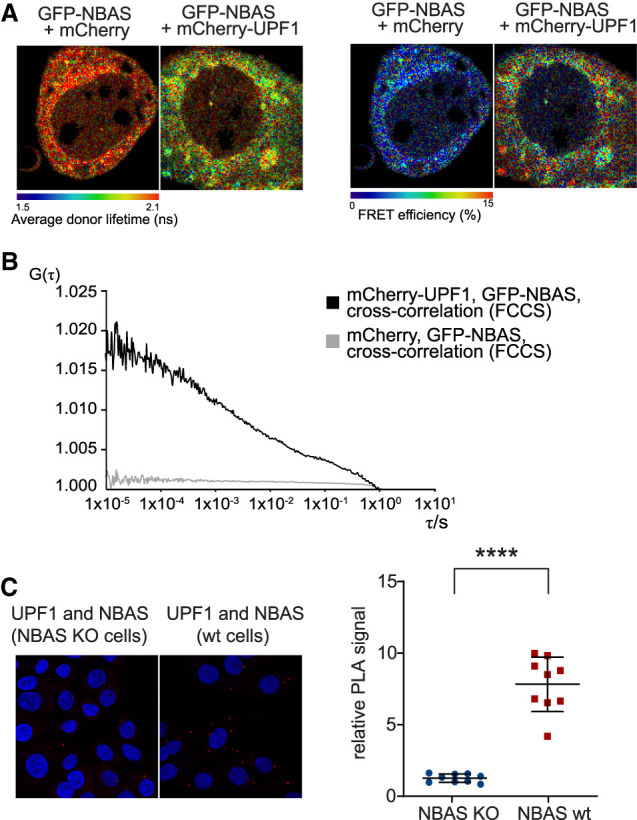
NBAS and UPF1 interact directly. (*A*) The interaction between NBAS and UPF1 was measured by FRET-FLIM. The average fluorescence lifetime of GFP-NBAS donor molecules was measured in HeLa cells expressing GFP-tagged NBAS together with mCherry-UPF1 or mCherry, as a control. Cells were pseudocolored by average lifetime (ns) where red represents longer lifetime and green corresponds to a shorter lifetime. (*Right* panel) Average fluorescence lifetime was used to calculate the FRET efficiency. Cells were pseudocolored by FRET efficiency (percentage). (*B*) The interaction between NBAS and UPF1 was measured by fluorescence cross-correlation spectroscopy (FCCS) in HeLa cells transiently expressing GFP-NBAS and mCherry-UPF1 or GFP-NBAS and mCherry, as a control. Increased G(τ) amplitude of the average cross-correlation curves corresponds to higher cross-correlation (higher fraction of codiffusing molecules) of GFP-NBAS and mCherry-UPF1 (in black), as compared with GFP-NBAS and mCherry average cross-correlation (in gray). (*C*) A direct interaction between endogenous NBAS and UPF1 was determined by PLA using antibodies against endogenous UPF1 and NBAS proteins in HeLa cells. The PLA signal in wild-type (WT) HeLa cells was compared with a HeLa NBAS knockout (KO) cells, as a negative control. The PLA signal was quantified in the graph where each point represents mean PLA count in one captured frame, relative to NBAS KO negative control. Significance was determined by two-tailed Mann-Whitney test. (****) *P* < 0.0001.

We used Förster resonance energy transfer (FRET) to probe the interaction of NBAS and UPF1 in HeLa cells in culture. This approach is based on the transfer of energy from the excited state of a donor fluorophore to an adjacent acceptor fluorophore when the two molecules are in the correct orientation and <10 nm apart. FRET was detected by a reduction in the amount of energy that the donor releases as fluorescence, measured by fluorescence lifetime imaging microscopy (FLIM) (Day et al. 2001; Ellis et al. 2008). Cotransfection of GFP-NBAS and mCherry-UPF1 in HeLa cells resulted in a reduction of the average donor fluorescence lifetime and an increase in the FRET efficiency, as compared with transfection of GFP-NBAS and mCherry (Fig. 5A). This experiment strongly suggests that NBAS and UPF1 interact directly. Next, we performed fluorescence cross-correlation spectroscopy (FCCS) to probe the interaction of NBAS and UPF1 in HeLa cells transiently expressing GFP-NBAS and mCherry-UPF1 or GFP-NBAS and mCherry, as a control. The cross-correlation signal is a direct indication of both molecules moving together, where a low amplitude of cross-correlation signal indicates that the labeled molecules diffuse separately, whereas high amplitude of cross-correlation signal is only achieved when both molecules are bound and diffuse together. The high cross-correlation signal observed in HeLa cells transiently expressing GFP-NBAS and mCherry-UPF1 indicated that NBAS and UPF1 interact directly (Fig. 5B, black curve), in contrast to the low amplitude of cross-correlation signal observed in cells expressing GFP-NBAS with mCherry, as a negative control (Fig. 5B, gray curve). Finally, we confirmed the interaction of endogenous NBAS and UPF1 using PLA. We observed a prominent PLA signal using antibodies against endogenous UPF1 and NBAS proteins, which was absent in cells that do not express NBAS (NBAS KO) (Fig. 5C). These results robustly show that NBAS and UPF1 interact directly in situ.

### NBAS recruits UPF1 to the membrane of the ER

**Figure 6.**
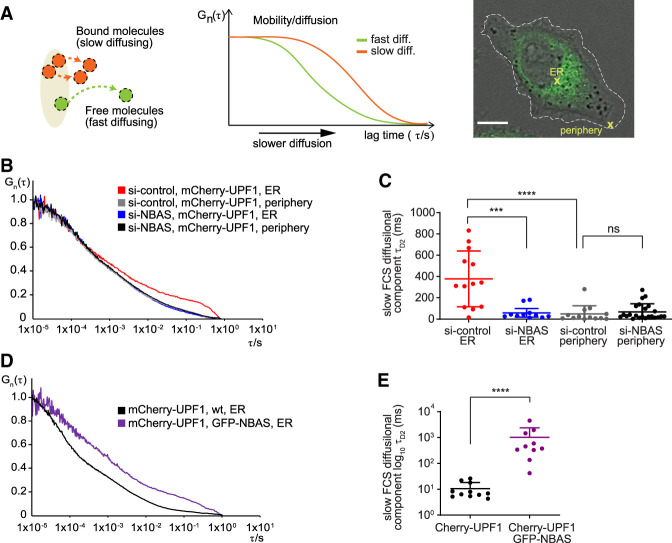
NBAS recruits UPF1 to the ER. (*A*) Schematic representation of the principle of fluorescence correlation spectroscopy (FCS). See the Materials and Methods for details. (*B*) Average autocorrelation curves of mCherry-UPF1 at the ER or cell periphery in the presence (si-control) or absence of NBAS (si-NBAS) are displayed. (*C*) Graph showing the derived characteristic decay times τ_D2_ (slow FCS component) of mCherry-UPF1, after fitting of the autocorrelation curves with a two-component model for diffusion (plus triplet correction). Significance was determined by the two-tailed Mann-Whitney test. (****) *P* < 0.0001; (***) *P* < 0.001; (ns) not significant. (*D*) Average autocorrelation curves of mCherry-UPF1 at the ER with physiological (WT) or increased (GFP-NBAS) level of NBAS expression. (*E*) Characteristic decay times τ_D2_ (slow FCS component), derived as in *C* of mCherry-UPF1 at the ER. Significance was determined by the two-tailed Mann-Whitney test. (****) *P* < 0.0001. Data showing FCS control experiments are in Supplemental Figure S8.

**Figure 7.**
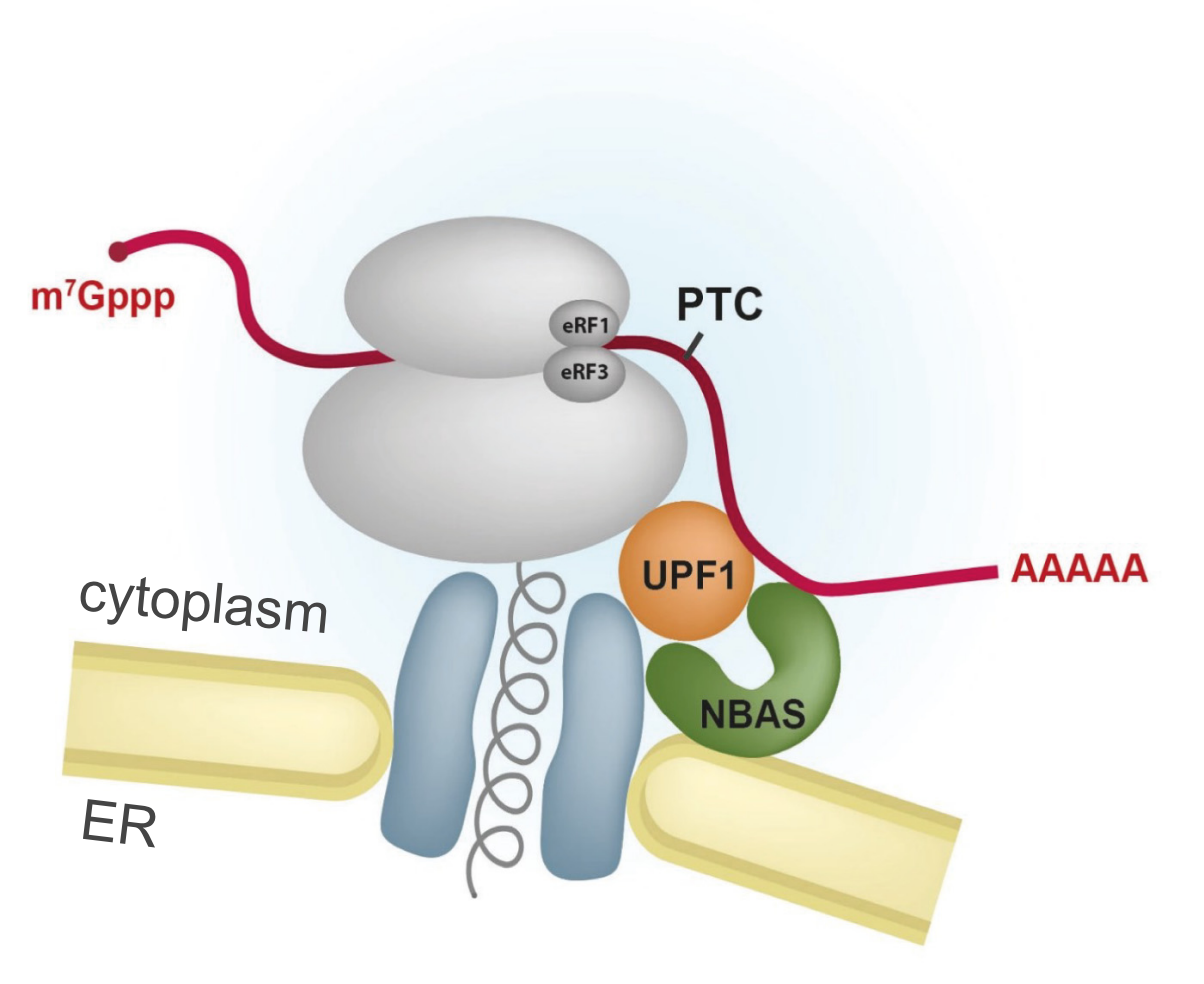
Model for an ER–NMD pathway. NBAS is an NMD factor that localizes to the membrane of the endoplasmic reticulum (ER). NBAS recruits the core NMD factor UPF1 and tethers it to the membrane of the ER to activate a local NMD response that targets for degradation mRNAs that are translated at the ER.

To probe the functional consequences of the NBAS–UPF1 interaction in vivo we used fluorescence correlation spectroscopy (FCS). This is a noninvasive method with single-molecule sensitivity that allows the analysis of the dynamic behavior of fluorescent molecules with high temporal resolution and at low, physiologically relevant concentrations in live cells (Liu et al. 2015; Papadopoulos et al. 2019). When the recorded fluorescence intensity fluctuations are caused by molecular movement, FCS measurements can be used to measure molecular mobility/diffusion rate of fluorescent molecules in a subfemtoliter detection volume in live cells. Thus, free, fast diffusing molecular movement is reflected by autocorrelation curves that display decays in shorter characteristic times (Fig. 6A, green curve), whereas bound or slow diffusing molecules are characterized by autocorrelation curves with slower decay times (Fig. 6A, orange curve). We performed FCS measurements to derive the mobility of transiently transfected mCherry-UPF1, or mCherry control at the ER, in the perinuclear region of the cell, or in the cytoplasm at the cell periphery, in HeLa cells (Fig. 6A, right panel). We observed that the mobility of mCherry-UPF1 in the cell periphery was very similar, irrespective of the levels of NBAS protein (Fig. 6B, cf. si-control, mCherry-UPF1, and periphery [in gray] with si-NBAS, mCherry-UPF1, and periphery [in black]). In contrast, a significantly slower mobility of mCherry-UPF1was observed at the ER in control cells bearing physiological levels of NBAS, as reflected by a marked shift of the autocorrelation curve toward longer characteristic times (Fig. 6B, red line). Depletion of NBAS increased the mobility of mCherry-UPF1 at the ER (Fig. 6B, blue line), so that the diffusion time of mCherry-UPF1 was uniform within the cell. In contrast, the mobility of mCherry alone was uniform throughout the cell and was not affected by the level of NBAS expression (Supplemental Fig. S8A,B). Fitting of the autocorrelation curves with a two-component diffusion model revealed that the characteristic decay time τ_D2_ of mCherry-UPF1 was considerably longer at the ER in the presence of NBAS (Fig. 6C, in red), whereas NBAS depletion caused significant reduction of the τ_D2_ mCherry-UPF1 decay times (Fig. 6C, in blue). The τ_D2_ decay time of mCherry-UPF1 was markedly shorter at the cell periphery than at the ER and was not affected by the depletion of NBAS (Fig. 6C). The observed reduced mobility of mCherry-UPF1 at the ER strongly suggests that UPF1 is part of a slower diffusing protein complex, and that NBAS is required for the retention of UPF1 at the ER. In agreement, overexpression of NBAS led to even slower mCherry-UPF1 mobility at the ER (Fig. 6D, cf. WT NBAS level, mCherry-UPF1, and ER [in black] with overexpressed GFP-NBAS, mCherry-UPF1, and ER [in purple]). In accordance, the characteristic decay time τ_D2_ of mCherry-UPF1 was significantly increased by the addition of GFP-NBAS (Fig. 6E). These results show that ER-localized NBAS acts to recruit UPF1 to the ER to activate a localized NMD response (Fig. 7).

## Discussion

### An NMD response at the ER

The targeting of mRNAs to specific subcellular sites for local translation has an important role in many cellular processes and during development (Decker and Parker 2006). In particular, translation of secreted and integral membrane proteins occurs on ER-bound ribosomes, whereas cytosolic protein synthesis mostly occurs on ribosomes dispersed throughout the cytoplasm. A subset of transcripts that are targeted for translation at the ER encode a leading signal peptide that is recognized after emerging from the ribosome by the signal recognition particle (SRP). These SRP-bound transcripts are subsequently targeted to the ER, and the resulting translated peptides are translocated across or inserted into the membrane by the SEC61 translocation channel (Walter and Johnson 1994). However, recent evidence showed that mRNAs encoding cytosolic proteins can also be translated by ER-associated ribosomes, suggesting a more diverse role for the ER in mRNA translation (Jagannathan et al. 2014b; Reid and Nicchitta 2015).

Despite the ER representing a specialized environment for the translation of a large proportion of cellular mRNAs, it remains unclear how RNA quality control pathways in general, and NMD in particular, operate on those mRNAs. Since NMD is dependent on active translation, transcripts coding for secreted and integral membrane proteins will not have sufficient exposure to the NMD quality control in the cytoplasm due to the intrinsic nature of ER-associated translation, which is spatially and temporally distinct from mRNA translation in the cytosol. In fact, ER targeted transcripts are only translationally active once they encounter the ER membrane (Wu et al. 2016). Thus, ER targeted transcripts will fail to undergo NMD in the cytoplasm, until their translation is activated at the ER membrane. We propose that these transcripts are instead targeted for degradation by an ER–NMD dedicated pathway that is responsible for the quality control of cellular mRNAs that are translated at the ER.

We showed previously that NBAS together with core NMD factors regulates the stability of a large number of endogenous RNA targets that are preferentially linked to cellular stress and membrane trafficking in nematodes, zebrafish, and human cells (Anastasaki et al. 2011; Longman et al. 2013). We also showed that NBAS contributes to a negative feedback regulatory network, in which the NMD pathway controls the levels of transcripts encoding NMD factors (Huang et al. 2011; Yepiskoposyan et al. 2011; Longman et al. 2013). Here, we used RNA-sequencing to profile changes in mRNA abundance upon depletion of NBAS or UPF1. Importantly, this analysis revealed that a large proportion of NBAS regulated targets were coregulated by UPF1, establishing them as candidate NMD targets (Fig. 2). These results, together with evidence provided by FCS experiments demonstrates that NBAS, which is anchored at the membrane of the ER, promotes the recruitment of the core NMD factor UPF1 and thereby activates the NMD response at the ER (Fig. 6). The decay step in NMD involves the recruitment of nucleases along two pathways that involve endonucleolytic decay mediated by SMG6 (Huntzinger et al. 2008; Eberle et al. 2009), or alternatively exonucleolytic decay mediated by the SMG5–SMG7 heterodimer (Loh et al. 2013). Interestingly, despite NBAS being preferentially associated with hypophosphorylated UPF1 in the SURF complex, we also detected its interaction with SMG5, SMG6, and SMG7 (Supplemental Fig. S7), which are required for mRNA degradation at the later stages of NMD. Altogether, this suggests that the presence of several core NMD factors is required for an active NMD pathway in the vicinity of the ER. In summary, we identified a localized NMD response at the ER that acts on transcripts localized and translated at the ER, which we term ER–NMD (Fig. 7).

### Biological role of ER–NMD pathway

Recent evidence supports a role for NMD in modulating the ER stress response by ensuring appropriate activation of the unfolded protein response (UPR) (Goetz and Wilkinson 2017). This pathway is able to sense and respond to excessive amounts of misfolded proteins in the ER (Walter and Ron 2011). Interestingly, mRNAs encoding several UPR components are targeted by NMD, including the UPR sensor IRE1α, as well as ATF-4 and CHOP that are activated by PERK branch signaling. Thus, NMD can act to control the threshold of cellular stress that is necessary to activate the UPR (Gardner 2008; Karam et al. 2015; Sieber et al. 2016). As a consequence, appropriate level of NMD activity is required to protect cells from detrimental stress response activation. In particular, pathological UPR activation is recognized as a major causal mechanism in neurodegenerative diseases including Alzheimer's, Parkinson's, and prion diseases, which are associated with the accumulation of misfolded disease-specific proteins (Hetz and Mollereau 2014). A chronic activation of PERK/eIF2α-P signaling results in a sustained reduction of global protein synthesis rates in neurons, caused by the phosphorylation and inactivation of eIF2α, which blocks translation at the level of initiation. Inappropriate eIF2α phosphorylation contributes to disease pathogenesis, and is observed in patients’ brains, and in mouse models of protein-misfolding neurodegenerative diseases (Hoozemans et al. 2009; Moreno et al. 2012). Thus, limiting the chronic activation of the UPR could have a neuroprotective effect in neurodegenerative diseases.

We hypothesize that modulation of the activity of the ER–NMD pathway could be crucial to regulate the response to ER stress. It is tempting to speculate that increasing the activity of the ER–NMD pathway will have a neuroprotective effect by limiting excessive UPR signaling in neurodegeneration. Indeed, a protective role for UPF1 has already been shown in primary neuronal models of amyotrophic lateral sclerosis (ALS) and frontotemporal dementia (FTD) induced by overexpression of the RNA-binding proteins, TDP43 and FUS (Barmada et al. 2015). Moreover, NMD has been shown to protect against the effects of hexanucleotide repeat expansion in C9orf72 (C9-HRE) found in *Drosophila* and cellular models of ALS/FTD (Xu et al. 2019; Ortega et al. 2020).

In summary, we uncovered a localized NMD response at the ER, which provides spatial and temporal separation from cytoplasmic NMD acting on mRNAs that are translated at the ER. This could have important implications to protect cells from ER stress/dysfunction that results from the accumulation of aberrantly processed RNAs, leading to the production of truncated proteins and affecting cellular homeostasis. This could be of particular relevance for many disease processes, such as neurodegenerative diseases, where an exacerbated ER stress is observed. Future studies will aim to further characterize this novel ER–NMD pathway; investigating its physiological role, as well as the biological consequences of manipulating its activity.

## Material and methods

### NMD reporter assays

HeLa cells stably expressing HBB WT or HBB NS39 reporters were mock-depleted or depleted twice of UPF2, NBAS, STX18, and STX5, and harvested 5 d after the first depletion. Alternatively, HeLa HBB NS39/WT-expressing cells were treated with 2 µg/mL actinomycin D (Sigma A9415) to block transcription and/or 2 µg/mL Brefeldin A (Sigma B5936) to block secretion for 6 h. The stability of HBB reporters was determined by qRT-PCR. The fluorescence-based NMD assay was performed as described previously (Pereverzev et al. 2015). NMD activity was determined by quantitative measurement of red and green fluorescence using FACS BD LSR-Fortessa X-20 SORP with BD FACSDiva software. Gates were set using a nonfluorescent control. Compensation was applied in BD FACSDiva software using single-transfected cells. Green fluorescence expression (488-525/50) was analyzed by gating the single–cell population in SSC-H/SSC-A scatter plot, followed by debris exclusion gate in SSC-A/FSC-A scatter plot, followed by gating only the red fluorescent-positive cells in 405-450/50 (autofluorescence) and 561-610/20 dot plot. Gate settings were kept constant during the experiment. Data were analyzed with FlowJo software (version 10.6.0). Between 4500 and 12,000 red fluorescent cells were analyzed in each biological replica. NMD disruption was determined by the increase of green fluorescence in red cells, normalized to mock-depleted cells.

### Single-molecule (sm) RNA FISH

smRNA FISH was performed following the Stellaris RNA FISH protocol for adherent cells (http://www.biosearchtech.com/stellarisprotocols) using RNase-free reagents. Transcript-specific Quasar 570-labeled Stellaris probes were designed with the Stellaris RNA FISH probe designer (Biosearch Technologies, Inc.; http://www.biosearchtech.com/stellarisdesigner), and resuspended in TE buffer to make 12.5 µM probe stock. Probe sequences are listed in Supplemental Table S5. HeLa cells were depleted as described above, with the addition of mEmerald-ER-3 (Addgene plasmid 54082; http://n2t.net/addgene:54082; RRID: Addgene 54082) at 1 µg per well in six-well plates during the second round of depletions. Cells were seeded on high-precision coverslips (Marienfeld 0107052), washed twice with PBS, and fixed with 4% formaldehyde for 10 min at room temperature. Cells were permeabilized in 70% ethanol for at least 1 h and hybridization of probes was performed in a humidified chamber for 15 h at 37°C. Probe hybridization and all subsequent steps were carried out in the dark. Following hybridization, coverslips were washed as described in the protocol. Nuclei were counterstained with 5 ng/mL of DAPI in buffer A for 30 min. Cell outlines were determined by staining with the addition of 20 nM MemBright 640-nm dye (Idylle MCO-MEM-640-1902) in buffer B for 15 min at room temperature. Cells were fixed and permeabilized prior to probe hybridization; therefore, under these conditions, this dye stained not only the cell membrane, as determined previously (Collot et al. 2019), but also the entire cell. Alternatively, we also used immunofluorescence with anti-Sec61β antibody (Proteintech 15087-1-AP), which under these conditions also stained the entire cell. This information was then used to determine the cell outline, which is indicated by a yellow line in Figure 3, drawn using Fiji. Coverslips were mounted in VectaShield and sealed with nail polish.

### Proximity ligation assay (PLA)

PLA assay was performed following Duolink PLA fluorescence protocol (Sigma-Aldrich). Cells were grown, fixed, and permeabilized as for immunofluorescence. Coverslips were then incubated for 1 h with PLA block buffer provided with the Duolink in situ PLA probes at room temperature and incubated with primary antibodies. Coverslips were then washed three times with Duolink wash buffer A and Duolink probe incubation, ligation, and PLA signal amplification were performed using Duolink in situ detection reagent Red kit according to the manufacturer's instructions. In the last wash, coverslips were incubated with DAPI at 50 ng/mL in 0.01× wash buffer B for 5 min, mounted in VectraShield, and sealed with nail varnish.

### FRET-FLIM

HeLa cells transiently expressing GFP-NBAS with mCherry or mCherry-UPF1 were grown on slides for 48 h after transfection, fixed in 4% PFA for 10 min, washed in PBS, and mounted in VectaShield with DAPI to visualize cell nuclei. Fluorescence lifetime images were acquired on a Leica SP5 SMD confocal laser-scanning microscope fitted with a time-correlated single photon counting module (PicoHarp 300) using a 63/1.4 numeric aperture HCX PL Apo oil immersion objective lens, as previously described (Saleeb et al. 2019). The donor EGFP was excited using a tunable white light supercontinuum laser operating at 488 nm and pulsing at 40 MHz. Emission was detected with an external single-photon avalanche diode (MicroPhoton Devices). Single-pixel fluorescence lifetime analyses were carried out with SymPhoTime version 5.4.4 (PicoQuant).

### FCS and FCCS

FCS measurements were performed by recording fluorescence intensity fluctuations in a very small, approximately ellipsoidal observation volume element (OVE) (∼0.2 μm wide and 1 μm long) that is generated in HeLa cells by focusing the laser light through the microscope objective and by collecting the fluorescence light through the same objective using a pinhole in front of the detector to block out-of-focus light. The fluorescence intensity fluctuations caused by fluorescently labeled molecules passing through the OVE were analyzed using temporal autocorrelation analysis. HeLa cells were mock-depleted or depleted of NBAS in two rounds of depletions. During the second round of depletions cells were cotransfected with 1 µg of mCherry control or of mCherry-UPF1plasmids. For NBAS overexpression, cells were cotransfected with GFP-NBAS and mCherry or mCherry-UPF1 (1 µg of each). Prior to FCS/FCCS measurement, cells were grown overnight on chambered coverslips (μ-slide, eight-well, Ibidi) and the growth medium was replaced with L-15 medium (Leibovitz) (Sigma-Aldrich L1518) immediately prior to FCS/FCCS measurements. Where appropriate, ER was visualized by the addition of 1 µM ER tracker (green) dye (Thermo Fisher E34251). Measurements were made only in weakly expressing cells. Fluorescence microscopy imaging of HeLa cells and FCS measurements were performed on a PicoQuant modified Leica SP5 microscope, which features Avalanche PhotoDiodes that enable close to single-photon detection (Vukojević et al. 2008). Fluorescence intensity fluctuations were recorded for 50 or 100 sec. Autocorrelation curves were analyzed using the SymphoTime 64 software package (PicoQuant). Control FCS measurements to assess the detection volume were routinely performed prior to data acquisition, using dilute solutions (10 nM and 20 nM, respectively) of Alexa488 and Alexa568 dyes (Petrášek and Schwille 2008). The variability between independent measurements reflects the variability between cells, rather than imprecision of FCS measurements.

### Quantification and statistical analysis

For information about the number of replicates, the meaning of error bars (e.g., standard error of the mean) and other relevant statistical analysis, see the corresponding figure legend. For information about how data were analyzed and/or quantified, see the relevant section in the Materials and Methods and/or the figure legend. GraphPad Prism software was used for the statistical analysis in Figures 1, C and D; 3A; 4B; 5C; and 6, C and E, and Supplemental Figures S1, B and C; S4; and S5, A and B. RStudio was used for the statistical analysis in Figure 2, A, B, and D, and Supplemental Figures S2 and S3.

#### Data availability

All RNA-seq data have been deposited in the Gene Expression Omnibus (GEO) database under accession number GSE152437.

## Supplementary Material

Supplemental Material
