# Supplementary material for: Identification of a localized nonsense-mediated decay pathway at the endoplasmic reticulum
Source: Genes Dev. 2020 Aug 1;34(15-16):1075–88. doi: 10.1101/gad.338061.120 (PMC7397857; doi:10.1101/gad.338061.120)
Supplement: Supplemental Material [file supp_34_15-16_1075__index.html]

Identification of a localized nonsense-mediated decay pathway at the endoplasmic reticulum — Supplemental Material 

# Identification of a localized nonsense-mediated decay pathway at the endoplasmic reticulum

## Supplemental Material

- Supplemental\_Material.pdf
