# Supplementary material for: Identification of a localized nonsense-mediated decay pathway at the endoplasmic reticulum
Source: Genes Dev. 2020 Aug 1;34(15-16):1075–88. doi: 10.1101/gad.338061.120 (PMC7397857; doi:10.1101/gad.338061.120)
Supplement: Supplemental Material [file supp_gad.338061.120_Supplemental_Material_.pdf]

Supplemental Material

Identification of a localized nonsense-mediated decay pathway at the endoplasmic reticulum

Dasa Longman, Kathryn A. Jackson-Jones, Magdalena M. Maslon, Laura C. Murphy, Robert S. Young,[1] Jack J. Stoddart, Nele Hug, Martin S. Taylor, Dimitrios K. Papadopoulos, and Javier F. Cáceres

MRC Human Genetics Unit, Institute of Genetics and Molecular Medicine, University of Edinburgh, Crewe Road South, Edinburgh EH4 2XU, UK

[1]Present address: Centre for Global Health Research, Usher Institute, University of Edinburgh, Old Medical School, Edinburgh EH8 9AG, UK

[*]Corresponding author: javier.caceres@igmm.ed.ac.uk

[*Keywords:* nonsense-mediated decay (NMD); RNA quality control; UPF1; NBAS; ER stress, UPR]

Supplemental Material includes:

Supplemental Figures: S1-S8

Supplemental Tables: S1-S5

Additional Materials and methods

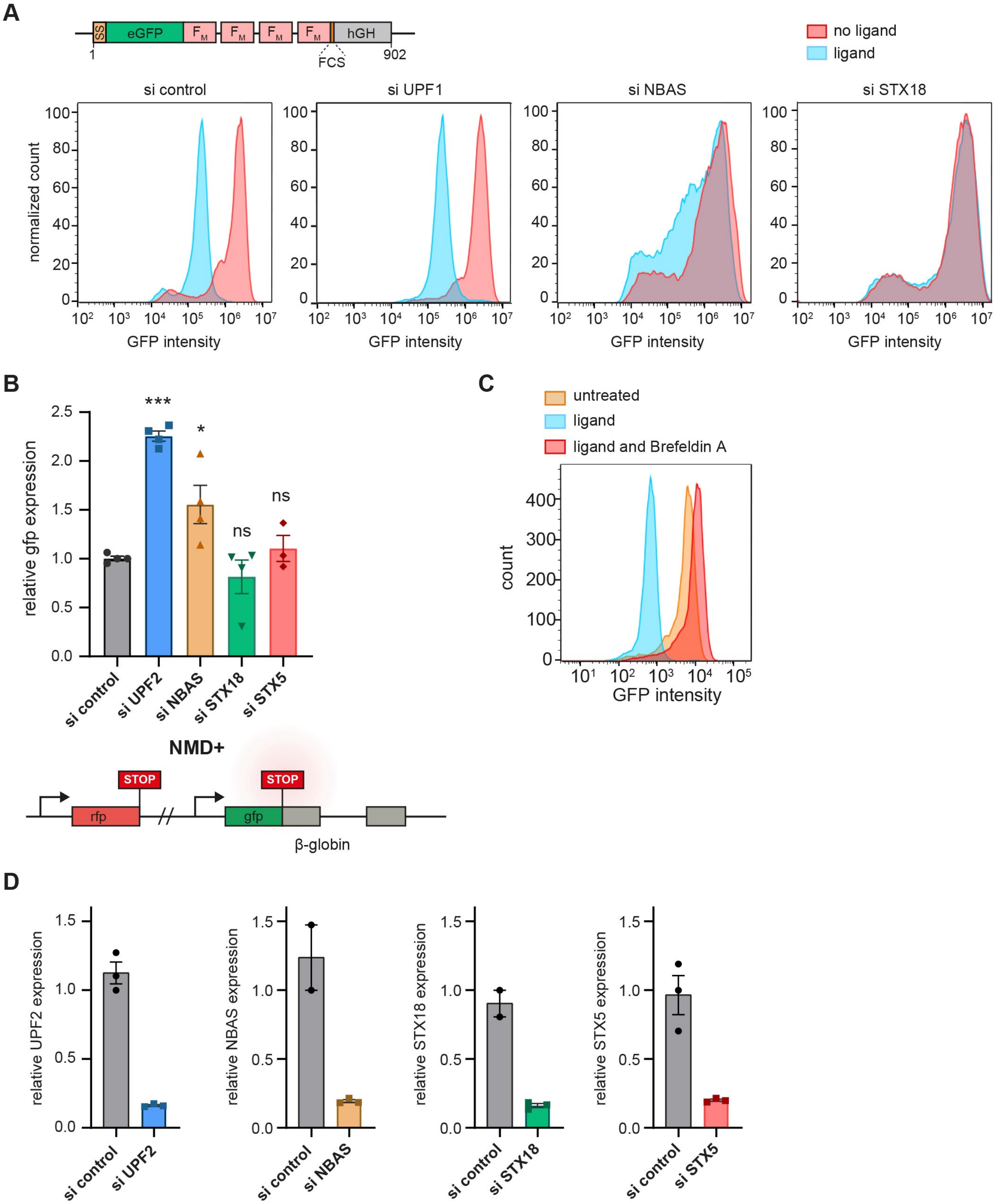
A
SS
eGFP
F M
hGH
1
902
FCS
no ligand
ligand
si control
si UPF1
si NBAS
si STX18
normalized count
GFP intensity
B
relative gfp expression
***
*
ns
ns
si control
si UPF2
si NBAS
si STX18
si STX5
NMD+
STOP
STOP
rfp
gfp
β-globin
C
untreated
ligand
ligand and Brefeldin A
count
GFP intensity
D
relative UPF2 expression
relative NBAS expression
relative STX18 expression
relative STX5 expression
si control
si UPF2
si NBAS
si STX18
si STX5

Supplemental Figure S1. Independent functions of NBAS in NMD and Golgi-to-ER transport, Related to Fig. 1. (*A*) Top panel shows a schematic representation of the GFP-based reporter expressed in HeLa C1 cells used to measure constitutive secretion. SS: signal sequence; eGFP: enhanced green fluorescent protein; FM: FKBP mutated; FCS: furin cleavage sequence; hGH: human growth hormone, and numbers represent amino acid residues. Defects in secretion are manifested by the inability of cells to lose GFP fluorescence even after addition of the ligand. Lower panels show examples of flow-cytometry before (in red), and after the addition of ligand (in blue), for control-depleted cells, cells depleted of NMD factors UPF1 and NBAS, and of secretion factor, STX18. (*B*) NMD activity is not affected by the depletion of secretion factors STX18 and STX5. HeLa cells were transfected with a fluorescent NMD+ reporter and depleted of NMD factors UPF2 or NBAS, or secretion factors STX18 or STX5. Depletion of both NMD factors increased the mean green fluorescence in comparison to mock-depleted cells, as expected, whereas STX18 and STX5 depletion did not affect the mean green fluorescence. Each point represents one biological replica, bars indicate mean with SEM. Significance was determined by two-tailed unpaired t-test: ***: P <0.0001; *: P <0.05; ns: not significant. (*C*) Brefeldin A treatment blocks constitutive secretion. HeLa C1 cells carrying a GFP-based secretion reporter were FACS-sorted to monitor their ability to secrete GFP. HeLa C1 cells (in orange) were able to secrete a GFP-based reporter following addition of ligand (in blue). Brefeldin A treatment led to a block in secretion and a concomitant accumulation of GFP even in the presence of ligand (in red). (*D*) Representative efficiency of depletion of NMD and secretion factors was determined by qRT-PCR relative to mock-depleted cells. In each case, relative expression of the depleted factor was normalized to POLR2J. Each point represents one biological replica, bars indicate mean with SEM.

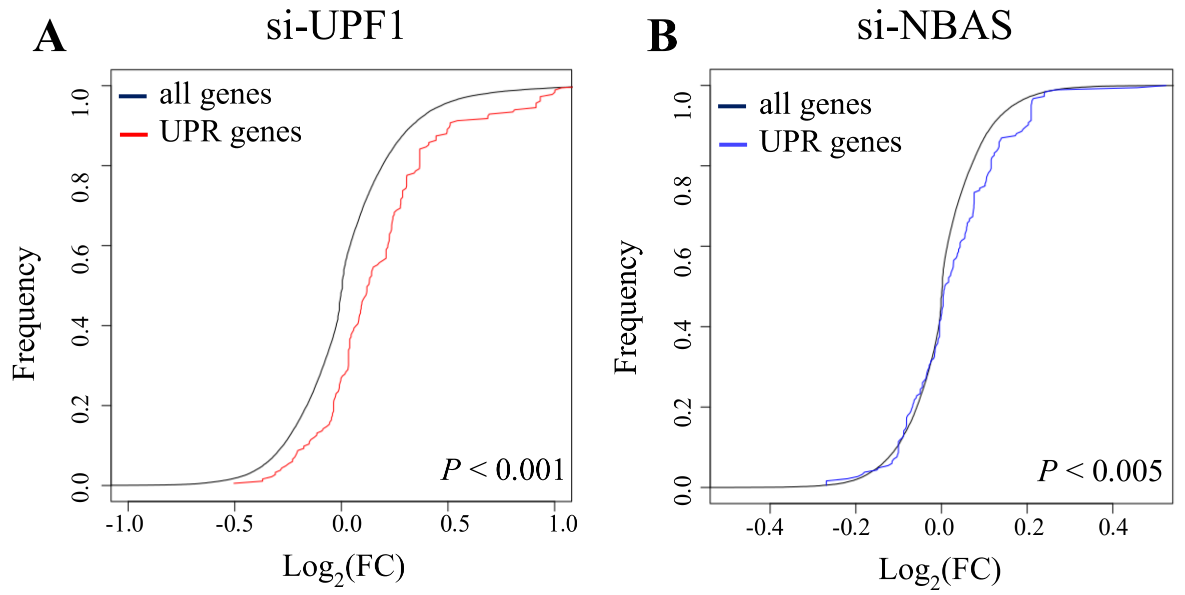

Supplemental Figure S2. Both NBAS and UPF1 regulate the ER stress response, Related to Fig. 2. Genes associated with the ER unfolded protein response (UPR) (GO: 0006986) show a significant increase in fold change compared to all genes, when either UPF1 (A) or NBAS (B) were depleted ($P < 0.001$ and $P < 0.005$, respectively; Wilcoxon rank sum test).

A

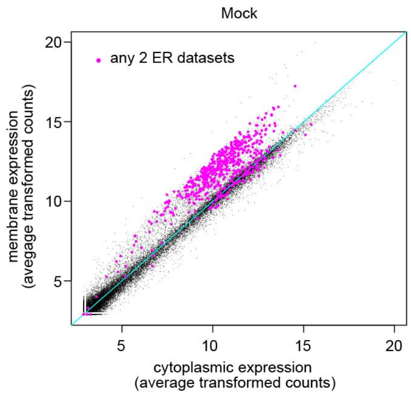

B

**n# membrane- associated genes**	**1999**	% regulated
up in siNBAS mem fraction	356	17.81%
up in siUPF1 mem fraction	788	39.42%
up in siUPF2 mem fraction	132	6.60%
**n# non-membrane genes**	**4194**	
up in siNBAS cyto fraction	147	3.51%
up in siUPF1 cyto fraction	1255	29.92%
up in siUPF2 cyto fraction	702	16.74%

C

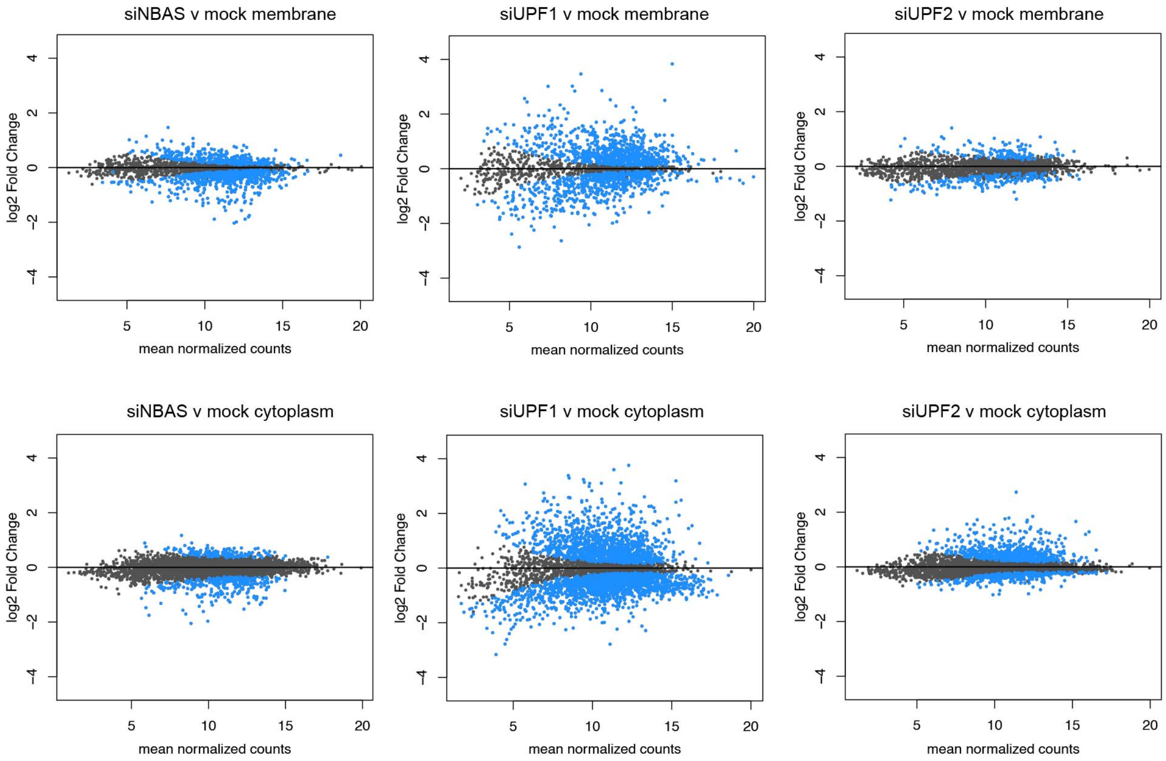

Supplemental Figure S3. NBAS regulates membrane-associated RNA targets, Related to Fig. 2. (*A*) Experimentally validated ER genes are present in the membrane fraction. Scatter plot of gene expression in membrane vs cytoplasmic fractions in mock-depleted cells, with experimentally validated ER genes present in at least 2 of the 3 ER datasets described in Fig. 2B are indicated in magenta. (Enrichment OR = 66.94, P <0.0001, Fisher's Exact test). (*B*) Summary table of the number of genes regulated by NMD factors in each cellular fraction. Genes were defined as membrane-

enriched or non-membrane-enriched as described in Fig. 2C. Genes were classified as regulated by the respective NMD factor if they were significantly (*P* <0.05) upregulated in that fraction, when the NMD factor was depleted. (*C*) Differential expression analysis of genes regulated by NMD factors in membrane fractions and in the cytoplasm. MA plots show log2 fold-change of gene expression plotted over mean of normalized counts (gene expression). Significantly changed genes (*P* <0.05) are indicated in blue.

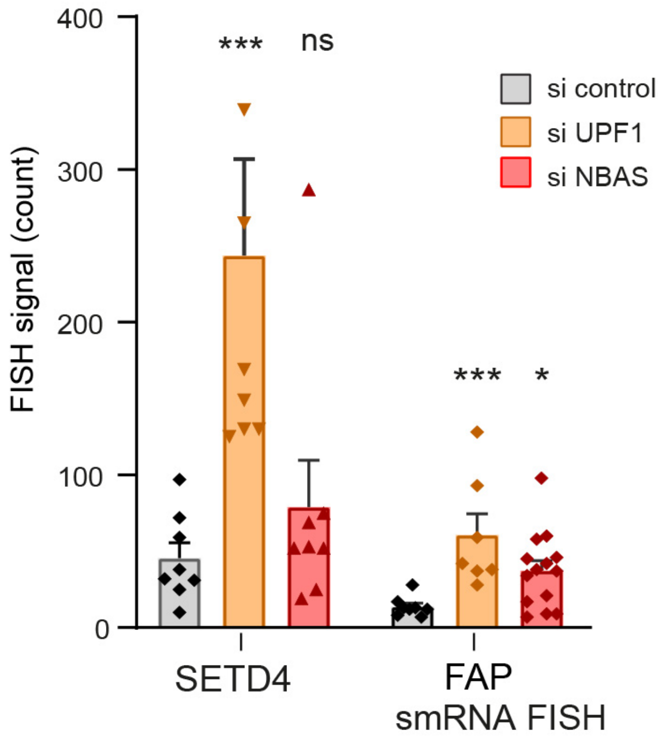

Supplemental Figure S4. Quantitation of *SETD4* and *FAP* transcripts upon UPF1 and NBAS depletion in smRNA FISH experiments, Related to Fig. 3. Relative changes in the expression of a cytoplasmic NMD target (SETD4) or an ER-localized NMD target (FAP) were determined by counting discreet FISH signal in individual cells. The FISH signal of FAP upon UPF1 or NBAS depletion is likely underestimated, as clusters of FISH dots were counted as one. Each point represents counts in one cell, bars indicate mean with SEM. Significance was determined by Mann-Whitney test: ***: P <0.001; *: P <0.05; ns: not significant.

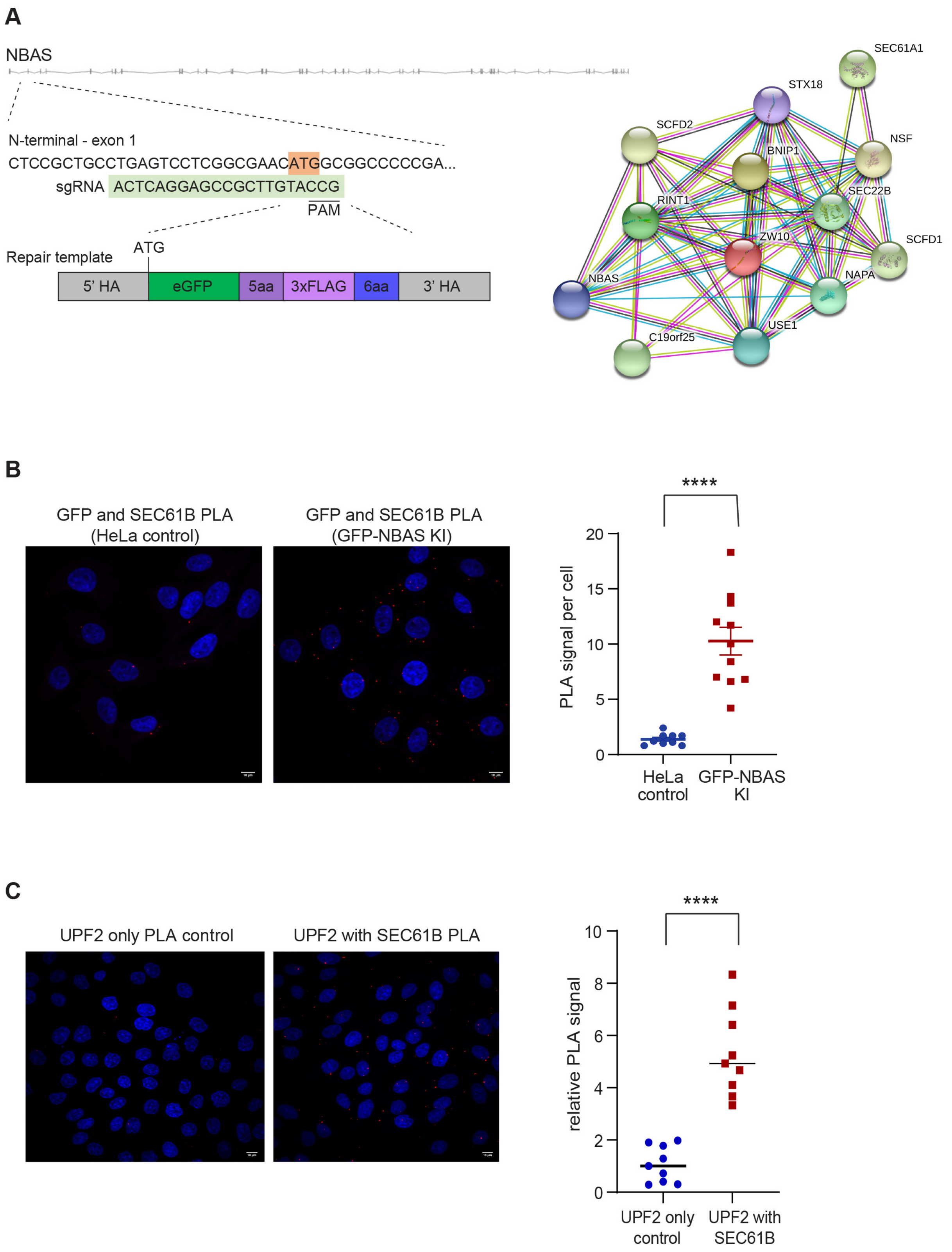
A
NBAS
N-terminal - exon 1
CTCCGCTGCCTGAGTCCTCGGCGAACATGGCGGCCCCCGA...
sgRNA ACTCAGGAGCCGCTTGTACCG
PAM
ATG
Repair template
5' HA
eGFP
5aa
3xFLAG
6aa
3' HA
SEC61A1
STX18
SCFD2
NSF
BNIP1
RINT1
SEC22B
ZW10
SCFD1
NAPA
NBAS
USE1
C19orf25
B
GFP and SEC61B PLA (HeLa control)
GFP and SEC61B PLA (GFP-NBAS KI)
****
PLA signal per cell
0
5
10
15
20
HeLa control
GFP-NBAS KI
C
UPF2 only PLA control
UPF2 with SEC61B PLA
****
relative PLA signal
0
2
4
6
8
10
UPF2 only control
UPF2 with SEC61B

Supplemental Figure S5. Localization of endogenous NBAS and UPF2 proteins to the ER, Related to Fig. 4. (*A*) NBAS is part of the Syntaxin 18 complex. Left panel depicts the strategy used to knock-in an eGFP/3xFLAG tag at the N-terminus of the *NBAS* locus, using CRISPR/Cas9-mediated genome editing in HeLa cells. Right panel shows the top NBAS interacting partners, and SEC61A1, identified by Mass spectrometry of GFP-pulled down cell lysates. The protein-protein interaction cluster was generated by STRING (https://string-db.org). The full list of proteins identified by Mass Spectrometry is presented in Supplemental Table T4. *(B)* Direct interaction between endogenous epitope-tagged NBAS and SEC61B proteins was determined by PLA using anti-GFP and anti-SEC61B antibodies, in HeLa KI (epitope tag knock-in) cells or wild-type HeLa cells, as a control. The graph shows the quantification of the PLA signal. Each point represents mean PLA count per cell in one captured frame, relative to the HeLa negative control. Significance was determined by Mann-Whitney test: ****: $P < 0.0001$. (*C*) UPF2 is localized in the close proximity of the SEC61 translocon component at the ER. Direct interaction between endogenous UPF2 and SEC61B proteins was determined by PLA using the anti-UPF2 and anti-SEC61B antibodies, in HeLa cells. The graph shows the quantification of PLA signal. Each point represents mean PLA signal per cell in one captured frame, relative to the HeLa negative control. Significance was determined by Mann-Whitney test: ****: $P < 0.0001$.

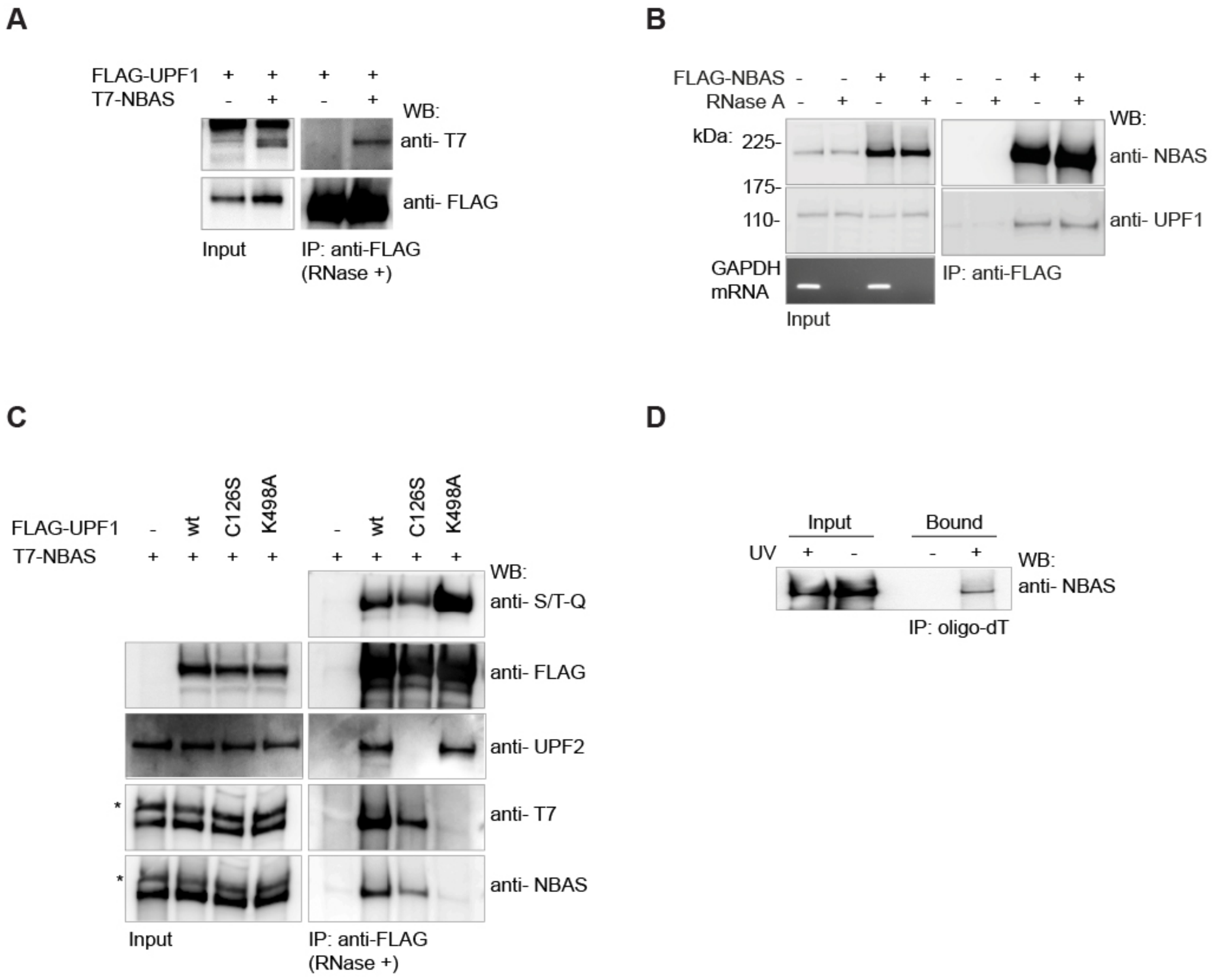

Supplemental Figure S6. NBAS interacts with UPF1 and preferentially associates with the SURF complex, Related to Fig. 5. (*A*) T7-tagged NBAS was co-expressed with FLAG-tagged UPF1 in HeLa cells. Cell lysates were subjected to Immunoprecipitation (IP) with anti-FLAG antibody in the presence of RNase. The NBAS-UPF1 interaction was revealed by Western Blot with anti-T7 antibody. (*B*) Interaction of endogenous UPF1 with FLAG-tagged NBAS in the absence or presence of RNase A. FLAG-tagged-NBAS was transiently expressed in HEK293T cells and immunopurified with anti-FLAG antibody. Co-immunopurification of endogenous UPF1 was analyzed by Western

Blot with anti-UPF1 antibody. RNA digestion was confirmed by RT-PCR for GAPDH. (*C*) T7-NBAS was co-expressed in HeLa cells with FLAG-tagged wild-type UPF1; C126S hypo-phosphorylated mutant UPF1 predominantly present in the SURF complex, or with K498A ATP-binding mutant UPF1, which is mainly associated with the DECID complex. Anti-FLAG IPs were performed in the presence of RNase and subjected to Western Blot analysis with the indicated antibodies. To detect phosphorylated UPF1, anti-FLAG-IPs were probed with a phospho-(Ser/Thr) ATM/ATR substrate antibody (anti-S/T-Q). (*D*) NBAS is bound to mRNAs. Hela cells were UV crosslinked and mRNP complexes were purified using oligo (dT) beads. The presence of NBAS in mRNP complexes was revealed by Western Blot with anti-NBAS antibody.

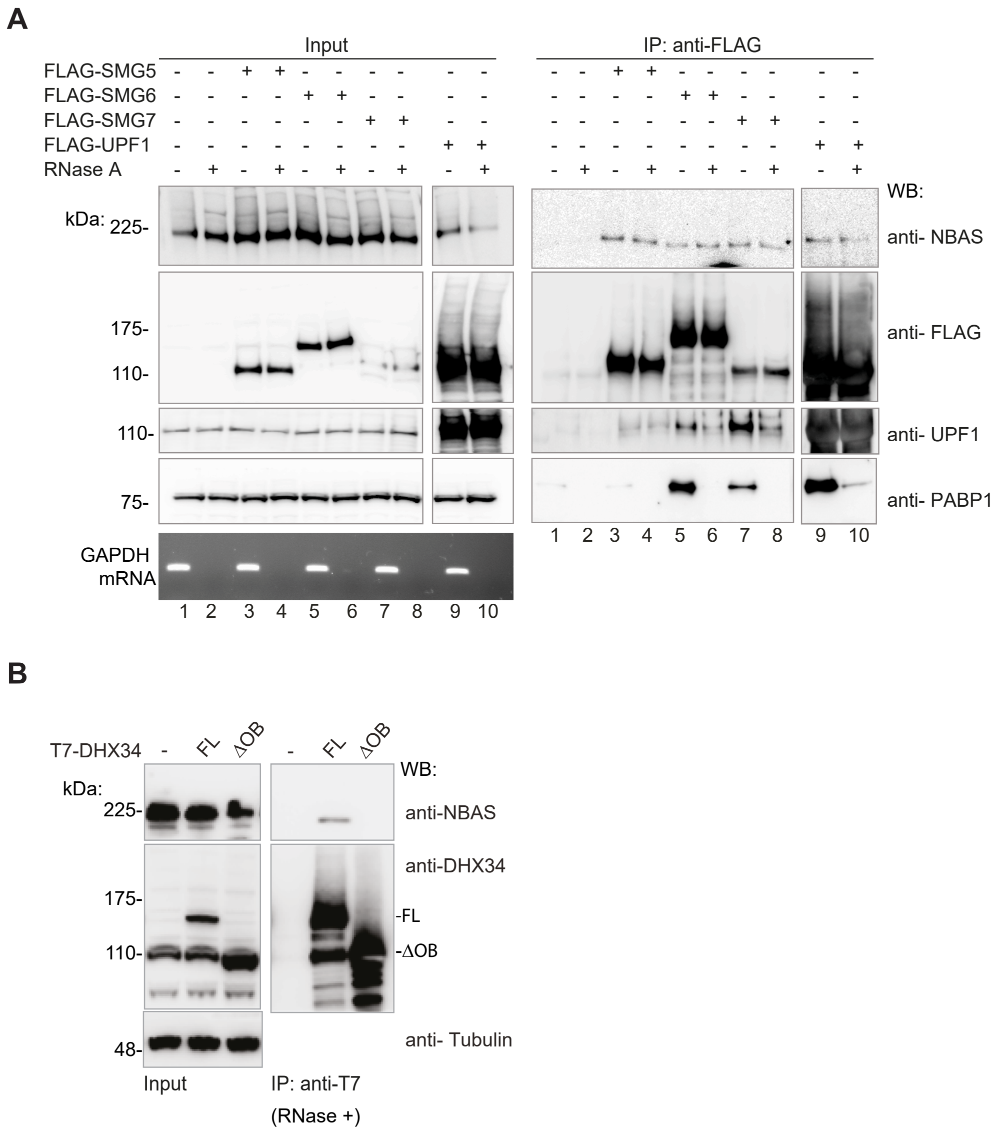

Supplemental Figure S7. Interaction of endogenous NBAS with core NMD factors, SMG5, 6, 7, UPF1 and DHX34, Related to Fig. 5. (*A*) Endogenous NBAS interacts with several NMD factors independently of RNA. HEK293T cells were transiently transfected with FLAG-tagged SMG5, SMG6, SMG7 and UPF1. Anti-FLAG Immunoprecipitations (IPs) were performed in the absence or presence of RNase A. Inputs and anti-FLAG IPs were subjected to Western Blot (WB) analysis with the indicated antibodies. The interaction with poly(A)-binding protein (PABP1) was used as a control

for the RNase treatment. Successful RNA digestion of the cell lysate was further confirmed by RT-PCR for the GAPDH housekeeping gene and analyzed on agarose gels. (*B*) T7-tagged full-length DHX34 interacts with endogenous NBAS. HEK293T cells were transfected with full-length (FL) and a truncated version of DHX34, lacking its OB-like domain (ΔOB). T7-DHX34 (FL and ΔOB) were immunopurified with anti-T7 antibody in the presence of RNase A. Inputs and T7-IPs were analyzed with anti-NBAS and anti-DHX34 antibodies. Anti-Tubulin antibody served as a loading control.

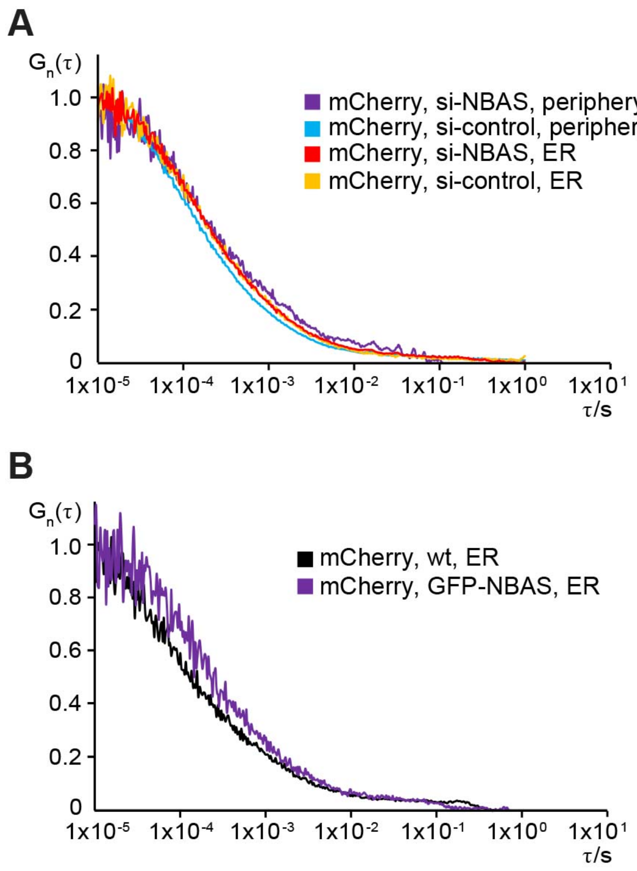

Supplemental Figure S8. FCS control experiments, Related to Fig. 6. (*A*) Average autocorrelation curves obtained for FCS measurements indicate that mCherry alone shows an almost complete overlap of decay times upon depletion of NBAS by RNAi, or between the ER and the cytoplasmic periphery. (*B*) Overexpression of GFP-NBAS does not affect the mobility of mCherry control at the ER.

Supplemental Table S1. Gene expression analysis.

Supplemental Table S2. GO term analysis of data presented in Supplemental Table S1.

Supplemental Table S3. Gene expression analysis of membrane and cytoplasmic fractions.

Supplemental Table S4. IP-Mass spectrometry protein interactors of an endogenously-tagged NBAS cell line.

Supplemental Table S5. List of oligonucleotides, smRNA FISH probes and siRNAs.

Additional materials and methods

Cell Culture and transfections.

HeLa cells were maintained in DMEM media with high glucose, GlutaMAX™ Supplement, pyruvate (Gibco Life technologies; 10569010) supplemented with 10% FCS, at 37°C in the presence of 5% CO_2. HeLa C1 cells and 3xFLAG-eGFP-NBAS cells were maintained in the same media with the addition of 1μg/ml of Puromycin. Cells were grown without antibiotic prior to transfections, which were carried out in Opti-MEM reduced serum medium (Gibco, 31985047). Transfections of siRNA oligos were done using DharmaFECT 1 (Dharmacon, T-2001-03) following manufacturer's protocol (Supplemental Table S5). Co-transfection of plasmids and siRNA oligos was achieved using Lipofectamine 2000 (ThermoFisher Scientific, 11668019) following manufacturer's instructions. For depletions, cells were plated in 12-well plates and transfected with 30 pmol of specific siRNAs. Cells were then expanded into 6-well plates and transfected with 50 pmol of the same siRNAs on the third day and were harvested for analysis on day 5 after the first depletion. For fluorescence based NMD assays HeLa cells were treated as above, with the addition of 50 ng NMD+ reporter that was co-transfected with siRNAs during the second round of depletion. For total RNA-sequencing and subcellular fractionation, cells were plated in 6-well plates and transfected with 30 pmol of indicated siRNAs. Cells were expanded into 10 cm plates the following day and were transfected with 30 pmol of the same siRNAs on day 3 and were harvested for analysis on day 4 after the first depletion.

Antibodies

Anti-UPF1, Bethyl # A300-036A, RRID:AB_203272; Anti UPF1, Bethyl # A300-038A, RRID:AB_2288326; Anti-GFP, Roche # 11814460001, RRID:AB_390913; Anti-Sec61β, Proteintech # 15087-1-AP, RRID:AB_2186411; Anti-NBAS, Abcam # Ab122370,

RRID:AB_11129367; Anti-UPF2, Santa Cruz Biotechnology # sc-374230, RRID:AB_10988267; Anti-Calnexin, Enzo Life Sciences # ADI-SPA-860-D, RRID:AB_2038898; Anti-T7, Novagen # 69522; Anti-FLAG, Sigma-Aldrich # F3165, RRID:AB_259529; Anti-Phospho-(Ser/Thr) ATM/ATR Substrate (anti-S/T-Q), Cell Signalling # 2851, RRID:AB_330318; Anti-Tubulin, Sigma-Aldrich # 4026, RRID:AB_477577, Anti-PABP1 PABP1 (4992, Cell Signaling), Anti- DHX34 is a peptide-specific antibody raised against human DHX34 obtained from Eurogentec (Hug and Cáceres 2014).

Flow cytometry-based assay for measuring constitutive secretion

The secretion assay was performed as previously described (Gordon et al. 2010). HeLa C1 cells constitutively express a secreted eGFP reporter fused to mutant FKBP proteins (FK506-binding protein), which form large aggregates that cannot be secreted. The F36M substitution leads to formation of dimers that can be dissociated by ligand binding, leading to efficient secretion (Gordon et al., 2010). HeLa C1 cells mock-depleted or depleted of UPF1, UPF2, DHX34, SEC13, NBAS, p31, RINT, ZW10 and STX18 were incubated with 1μM D/D Solubilizer (ligand) (Clontech 6350540 for 1.5 h to facilitate secretion or left untreated for comparison. Cells were trypsinized, washed with cold PBS and placed on ice to stop further secretion. To measure the amount of GFP reporter remaining, cells were analyzed by FACS BD AccuriTM (488 nm excitation laser, FL-1: 533/30 nm emission filter) running the software BD AccuriTM C6. Gates were set using a non-fluorescent control. GFP expression was analyzed by gating the single cell population in SSC-H/SSC-A dot plot, followed by debris-exclusion gate in SSC-A/FSC-A dot plot. Between 5000 and 10000 single cells were analyzed for each sample. The mean GFP fluorescence was calculated with FlowJo™ Software (Version 10.6.0). The relative GFP fluorescence

remaining after secretion was calculated as a ratio between the mean fluorescence of depleted cells incubated with the ligand and depleted cells. The relative fluorescence remaining of untreated C1 cells was set to one, and the threshold for a secretion defect was set to two.

Cell fractionation

HeLa cells were mock-depleted or depleted twice of UPF1, UPF2 and NBAS as described above. Cellular fractionation was performed as described before (Jagannathan et al. 2011) with some modifications. Briefly, four days after the first depletion, cells were detached using trypsin and washed twice in 1ml of ice-cold PBS (500g, 10 min, 4°C). Cellular pellets were resuspended in 0.4 ml of permeabilization buffer (110 mM KOAc, 25 mM K-HEPES pH 7. 2, 2.5 mM $Mg(OAc)_2$, 1 mM EGTA, 0.015% digitonin, 1mM DTT, 1× Complete Protease Inhibitor Cocktail, 40 U/mL RNaseOUT™) and incubated for 5 min on a rotating wheel at 4°C. The resulting cytosolic fractions were recovered by centrifugation at 2000g for 10 minutes, 4°C. Cells were then lysed in 0.4 ml of NP-40 lysis buffer (400 mM KOAc, 25 mM K-HEPES pH 7.2, 15 mM $Mg(OAc)_2$, 1% (v/v) NP-40, 1 mM DTT, 1× Complete Protease Inhibitor Cocktail, 40 U/mL RNaseOut) for 30 min on ice. The membrane fraction was recovered by centrifugation at 7000g for 10 min at 4°C. Both cytosolic and membrane fractions were clarified by centrifugation at 7500g for 10 min at 4°C. Digitonin, DTT, Complete Protease Inhibitor Cocktail and RNaseOUT™ were added fresh to the buffers. RNA from both fractions was isolated using PureLink RNA Mini Kit (Life Technologies) according to manufacturer's instructions. DNA was removed using TURBO DNA-free™ DNase I kit (Invitrogen Ambion; AM1907).

Gene expression profiling: RNA extraction, library preparation and RNA-sequencing

Total RNA was isolated from depleted cells by phenol-chloroform extraction and treated with TURBO DNA-free™ DNase I kit (Invitrogen Ambion; AM1907). Libraries were prepared following NEBNext Ultra Directional RNA Library Prep Kit for Illumina (New England Biolabs; #E7420) and Agencourt AMPure XP Beads (Beckman Coulter; A63881). Samples were ligated to barcode primers 1-20 of NEB Next Index Primers for Illumina sets 1 and 2 (New England Biolabs) and libraries analyzed using DNA High Sensitivity chip on an Agilent 2100 Bioanalyzer before being pooled. 150 base pair, paired-end sequencing was performed using the S2 flow cell on the NovaSeq 6000 System (Illumina Inc.). Library molarity for sequencing was calculated using Qubit dsDNA quantification results and fragment size information from Bioanalyzer results. Sequencing was performed by Edinburgh Genomics, Edinburgh, UK.

RNA-sequencing analysis

FASTQ files were quality control checked for base and sequence quality scores, and adapter contamination using fastQC (v0.11.7; Babraham Bioinformatics). Reads were aligned using Spliced Transcripts Alignment to a Reference (STAR, v2.5.1b) (Dobin et al. 2013) or pseudoaligned using kallisto (v0.43.1; (Bray et al. 2016)). Kallisto index was created by combining "all basic gene annotation" and "long non-coding RNA gene annotation" fasta files, downloaded from Gencode (www.gencodegenes.org/releases/current.html), Release 27 (GRCh38.p10). Kallisto was run with 100 bootstraps. Reads per feature in STAR output files was counted using HTSeq (v0.9.1; ((Anders et al. 2015)). Abundance files were then analyzed for fold changes by running DESeq2 (v1.14.1) differential expression analysis (Love et al. 2014). Gene Ontology enRIchment anaLysis and visuaLizAtion (GOrilla) (http://cbl-gorilla.cs.technion.ac.il/) (Eden et al. 2009)) and Database for

Annotation, Visualization and Integrated Discovery (DAVID) (https://david.ncifcrf.gov/home.jsp) (Huang et al. 2009) were used for Gene Ontology (GO) analysis. To investigate genes linked to specific GO terms, genes were annotated using biomaRt Bioconductor R package (Durinck et al. 2005). For NBAS and UPF1 targets overlap with experimentally ER-localized genes, we first downloaded relevant supplementary data. For APEX-seq data (Fazal et al. 2019), ER genes were defined as all those labeled "ERM_Gene" and for ER Fractionation sequencing data (Reid and Nicchitta 2012) those genes that were labelled "ER". For ER Proximity-specific Ribosome Profiling data (Jan et al. 2014), gene names were converted from uniprot.protein.id to official gene symbols using DAVID (https://david.ncifcrf.gov/home.jsp) (Huang et al., 2009), and a 1.5-fold enrichment at ER cut off applied. For fractionated RNA-sequencing, gene expression in both fractions in control cells was compared by using variant stabilized transformation of tpm, averaged across replicates. Membrane-associated genes were defined as those >2-fold higher expression in membrane fraction than cytoplasmic fraction, all other genes were termed non-membrane. Membrane-enrichment was confirmed by overlap with three experimental ER datasets (see above). Changes in gene expression resulting from depletion of individual NMD factors was measured by percentage of 'membrane-associated' genes in membrane fraction, and of 'non-membrane' genes in cytoplasmic fraction regulated. Genes were determined as regulated if they were significantly (P <0.05) increased in expression when the relevant factor was depleted.

Quantitative RT-PCR

Total RNA was isolated using RNeasy Mini kit and resuspended in nuclease-free water at 100ng/µl. qRT–PCR was performed using SuperScriptIII One-Step RT–PCR Kit (Invitrogen) following the manufacturer's instructions. All RT-PCRs were run on the CFX96 Real-Time System (Bio-Rad

machine, following this program: RT at 50°C for 30 min, 95°C for 2 min, then 40 cycles of 95°C for 30 sec, 55°C for 20 sec, 70°C for 20 sec followed by the plate read step. Each sample was run in 3 technical replicates. Primers were designed using Roche Real-Time Ready Configurator and combined with Roche Universal Probe Library (See Supplemental Table S4). Gene expression data was analysed by the delta Ct method, with each gene normalised to housekeeping gene *POL2RJ*. Unpaired two-tailed t-test was used for statistical analysis.

Immunofluorescence

Cells were grown on coverslips, fixed with 4% paraformaldehyde at room temperature for 10 min, washed with PBS and permeabilized with 0.5% Triton X-100 at room temperature for 10 min. Coverslips were then incubated for 1 h with block buffer (1% BSA, 0.01% Triton X-100 in PBS), followed by primary antibodies (diluted 1:500 in block buffer) in a humidified chamber overnight at 4°C. Coverslips were washed 3 times with wash buffer (0.01% Triton X-100 in PBS). Secondary antibodies (Alexa Flour® 488 or Alexa Flour® 594, Molecular Probes, diluted 1:1000 in block buffer) were incubated with coverslips in a dark, humidified chamber for 1 h at room temperature. Coverslips were then washed 3 times with wash buffer and stained with 4,6-diaminidino-2-phenylidole (DAPI) at 50ng/ml, mounted in Vectashield (Vector) and sealed with nail varnish. For Digitonin treatment, media was aspirated, and cells incubated for 10 min on ice in cold PBS and later incubated in Digitonin buffer (110 mM KoAc, 25 mM K-HEPES, 2.5 mM $MgCl_2$, 1 mM EGTA) with 0.01% Digitonin for 5 min on ice, washed in room temperature PBS and fixed with 4% paraformaldehyde, as above.

Design and screening of CRISPR cell lines

The design of the guide RNAs (gRNAs) was undertaken, as previously described (Ran et al. 2013). gRNAs were designed using sgRNA Designer CRISPRko (Broad institute, https://portals.broadinstitute.org/gpp/public/analysis-tools/sgrna-design) and Cas-Designer (RGEN Tools, http://www.rgenome.net/cas-designer/). For the tagged-NBAS cell line, four gRNAs were selected by closest proximity to the start codon and highest predicted efficiency. For each guide RNA, the complementary sequence was determined and a BbsI restriction site added to both oligos. Designed gRNAs were ordered as custom single stranded DNA oligos with an extra G at the 5' end, and 5' phosphate at the reverse complement DNA strand (IDT). Top and bottom strands of gRNAs were annealed at a concentration of 100 μM and cloned into the px459 V2.0 vector using BbsI restriction cloning. 1 μl of a 1:250 dilution of annealed gRNAs was ligated with the T4 DNA Ligase (NEB) into 36 ng of the px459 V2.0 vector. To assess cutting efficiency of gRNAs, each plasmid was transfected into HeLa cells and RNA extracted as above. PCR over the target region was performed with custom primers (Supplemental Table S4) and the resulting products assessed by DNA electrophoresis. PCR products were also sent for Sanger sequencing and traces were analyzed by TIDE (REF) and ICE (REF) to give exact cutting efficiencies for each guide. Repair template was ordered as a custom plasmid from IDT on a pUCIDT-AMP backbone. For the generation of NBAS knock out (KO) HeLa cells, two guides (NBAS KO_A and NBAS KO_B) targeting the 5th exon of the NBAS gene were cloned into the pSpCas9n(BB)-2A-GFP vector encoding the (D10A nickase mutant (PX461), as described above. pSpCas9n(BB)-2A-GFP (PX461) (Addgene plasmid # 48140; http://n2t.net/addgene:48140; RRID: Addgene_48140). The gRNA/Cas9 plasmid and repair template plasmid were transfected into HeLa cells as described above. After 24 h, media was supplemented with 1.5 μg/ml puromycin to select for cells expressing the gRNA/Cas9 plasmid. Once control cells

were dead, surviving cells' fluorescence was measured by FACS and single cells deposited into wells of a 96-well plate. After approx. 2 weeks clonal expansion, genomic DNA was extracted by lysing with DirectPCR Lysis Reagent Cell (Peqlab, VWR) supplemented with 0.5 µg/µl Proteinase K (Invitrogen), at 55°C O/N. PCR of the N-terminal locus of NBAS was performed using previously mentioned custom primers and SYBR green Master Mix. PCR products were resolved on an agarose gel for genotyping. The correct repair template integration was validated by genomic DNA sequencing and expression of tagged-NBAS was validated by Western blotting. Screening for the NBAS KO clones was performed as above. HeLa cells co-transfected with NBAS KO_A and _B pSpCas9n(BB)-2A-GFP plasmids were FACS-sorted into 96-well plates 48 h after transfection. Growing colonies were screened by PCR of genomic DNA as described, using NBAS KO F and R primers. NBAS expression was assessed by Western Blotting and genomic DNA of the NBAS KO was checked by sequencing.

Image Capture and analysis

For smRNA FISH experiment, epifluorescent images were acquired using a Photometrics Coolsnap HQ2 CCD camera and a Zeiss AxioImager A1 fluorescence microscope with a Plan Apochromat 100x 1.4NA objective, a Nikon Intensilight Mercury based light source (Nikon UK Ltd, Kingston-on-Thames, UK) and either Chroma #89014ET (3 colour) or #89000ET (4 colour) single excitation and emission filters (Chroma Technology Corp., Rockingham, VT) with the excitation and emission filters installed in Prior motorized filter wheels. A piezoelectrically driven objective mount (PIFOC model P-721, Physik Instrumente GmbH & Co, Karlsruhe) was used to control movement in the z dimension. Step size for z stacks was set at 0.2 µm. Hardware control, image capture and analysis were performed using Nikon Nis-Elements software (Nikon UK Ltd, Kingston-on-Thames, UK).

Images were deconvolved using a calculated point spread function with the constrained iterative algorithm of Volocity (PerkinElmer Inc, Waltham MA). Image analysis was carried out using the FIJI/ImageJ software (2.0.0-rc-69/1.52p) (Schindelin et al. 2012). To measure the distribution of FISH signal within cells, a maximum intensity projection of each deconvolved z-stack was created and the DAPI signal was thresholded to create a mask of the nucleus. This mask was inverted and a euclidean distance map was created using the "Distance Map" function. This resulted in an image where all pixels that were inside the nucleus had a value of zero and the other pixels had values based on their distance from the closest nucleus edge (in pixels). To detect the centroids of FISH spots in the FISH signal channel, the ImageJ function "Find Maxima" was used with a prominence setting of 800. The x-y co-ordinates of each maximum point was measured on the distance map, giving a distance of the FISH spots to the edge of the nucleus. These values were converted to microns using the pixel spacing value of the original image. For immunofluorescence and PLA experiments images were acquired on a Nikon Confocal A1R confocal microscope using a Plan Apochromat 100x 1.4NA objective. The microscope comprises a Nikon Eclipse TiE inverted microscope with Perfect Focus System and is equipped with 405nm diode, 457/488/514 nm Multiline Argon, 561 nm DPSS and 638 nm diode lasers. Detection is via four Photomultiplier tubes (2x standard Photomultiplier tubes and 2x GaAsP PMTs). Data were acquired using NIS Elements AR software (Nikon Instruments Europe, Netherlands). Z-stacks of images were acquired with a 0.2 µm step, scan size 1024x1024, 1.2x zoom and 2x frame averaging. Image analysis was carried out using the FIJI/ImageJ software.

Mass Spectrometry

Cells were harvested and lysed as in immunoprecipitation protocol (see below). α-GFP antibody-coupled magnetic beads (Sigma) were equilibrated with IP buffer. Lysates were resuspended in 500

µl IP buffer for capture of GFP-NBAS bound proteins and subsequent mass spectrometry analysis. Immunoprecipitation was performed on Kingfisher Duo robot (Thermo) for 4 h. All steps were carried out at 4°C. Beads were then transferred for two washes in IP buffer and three washes in TBS (300 µl each). After transfer into 100 µl 2M urea, 100 mM Tris, 1 mM DTT containing 0.3 µg trypsin per sample, beads were incubated at 27°C for 30 min with mixing to achieve limited proteolysis. The beads were then removed, and tryptic digest of the released peptides was allowed to continue for 9 h at 37°C. Following this, peptides were alkylated by adding iodoacetamide to 50 mM and incubated at room temperature for 30 min. Finally, peptides were acidified by addition of 8 µl 10% TFA. An estimated 10 µg of the resulting peptide solution was loaded onto an activated (20 µl methanol), equilibrated (50 µl 0.1% TFA) C18 StAGE tip, and washed with 50 µl 0.1% trifluoroacetic acid (TFA). The bound peptides were eluted into a 96-well plate (Axygen, Corning Inc., Corning, NY, USA) with 20 µl 80% acetonitrile (ACN), 0.1% TFA and concentrated to less than 4 µl in a vacuum concentrator. The final volume was adjusted to 15 µl with 0.1% TFA. Mass spectrometry was carried out by IGMM Mass Spectrometry core facility. Online LC was performed using a Dionex RSLC Nano (Thermo Fisher Scientific). Following the C18 clean-up, 5 µg peptides were injected onto a C18 packed emitter and eluted over a gradient of 2%-80% ACN in 48 minutes, with 0.5% acetic acid throughout. Eluting peptides were ionised at +2.2kV before data-dependent analysis on a Thermo Q-Exactive Plus. MS1 was acquired with mz range 300–1650 and resolution 70,000, and top 12 ions were selected for fragmentation with normalised collision energy of 26, and an exclusion window of 30 seconds. MS2 were collected with resolution 17,500. Raw MS data were analyzed using MaxQuant (v 1.5.6.5) (Max Planck Institute of Biochemistry) in conjunction with UniProt human reference proteome release 2016_11 (uniprot.com), with match between runs (MS/MS not required), LFQ with 1 peptide required, and statistical analyses performed in R (RStudio 1.1.453 / R x64 3.4.4)

(rstudio.com) using Wasim Aftab's LIMMA Pipeline Proteomics (github.com/wasimaftab/LIMMA-pipeline-proteomics) implementing a Bayes-moderated method. Interactome analysis including gene ontology was carried out by inputting protein list into STRING (string-db.org/).

Immunoprecipitation and Western Blotting

Cells were washed and harvested in ice-cold PBS before pellets were lysed with immunoprecipitation (IP) buffer (20 mM Tris-HCl pH 8, 150 mM NaCl, 1mM EDTA, 1% NP-40, 0.2% Deoxycholate, Complete Protease Inhibitor (Roche), Phosphor STOP (Roche), 1 mM DTT) for 20 min on ice. Cell lysates were treated with 40–80 mg/ml RNase A per 1 ml of extract. Lysates were precleared with Dynabeads Protein-G (Novex, Life Technologies) for 1 h, rotating at 4°C. Anti-FLAG M2 antibody coupled magnetic beads (Sigma-Aldrich M8823) were washed x3 with IP buffer before incubation with the precleared lysate overnight rotating at 4°C. Beads were washed x5, each for 5 min with IP buffer and then bound protein was eluted by boiling in SDS sample buffer supplemented with reducing agent for 5 min. Proteins were resolved by SDS-PAGE using NuPAGE 3-8% Tris-Acetate precast gels (Novex, Life Technologies) run for ~1 h at 170V in 1x Tris-Acetate running buffer. Protein transfer was achieved using the iBlot™ 2 Gel Horizontal Transfer Device (Invitrogen). Nitrocellulose membranes were blocked in 5% BSA in PBS/Tween 20 (0.1%) for a minimum of 1h at room temperature and probed with the appropriate primary antibody diluted in blocking solution 1:3000. FLAG antibody was used at 1:10,000 dilution and T7 antibody was used at 1:5000 dilution. HRP-conjugated secondary antibodies (BioRAD) were used at 1:10,000 and blots developed with ChemiGlow detection reagent and visualized using ImageQuant LAS 4000 chemiluminescent camera (GE Healthcare).

In situ UV cross-linking mRNP capture assay

In situ UV cross-linking mRNP capture protocol was adapted from (Piñol-Roma and Dreyfuss 1992). Briefly, cells, grown in 150 mm plates, were irradiated with 0.15 Joule/cm^2 at 254-nm UV light, on ice, in ice-cold PBS. Cells were then scraped from plates in PBS and pelleted. Cells were lysed in 1ml of ice-cold lysis buffer (20 mM Tris-HCl (pH 7.5), 500 mM LiCl, 0.5% LiDS, 1 mM EDTA and 5 mM DTT), and then sonicated 30s on/30s off for 5 cycles at 4°C. After 10 min incubation at 4°C, the lysates were fractionated by centrifuge (15,000 xg, 4°C, 15 min), 10% sample was stored as input. 100 µl per sample oligo(dT) 25 magnetic beads were equilibrated in lysis buffer and incubated with lysate overnight at 4°C with rotation. Beads were washed once with 1ml of lysis buffer, twice with Buffer 1 (20 mM Tris-HCl (pH 7.5), 500 mM LiCl, 0.1% LiDS, 1 mM EDTA and 5 mM DTT), twice with Buffer 2 (20 mM Tris-HCl (pH 7.5), 500 mM LiCl, 1 mM EDTA and 5 mM DTT) and twice with Buffer 3 (20 mM Tris-HCl (pH 7.5), 200 mM LiCl, 1 mM EDTA and 5 mM DTT). All washes for 5 min at 4°C. Captured mRNPs were eluted from the beads with 100µl elution buffer (20 mM Tris-HCl (pH 7.5) and 1 mM EDTA) for 3 min at 55°C and treated with 200 U of RNase A in 500 µl of 10× RNase buffer, for 1h at 37 °C. Liberated mRNA binding proteins were mixed 1:1 with SDS PAGE sample buffer (1x LDS sample buffer, 1x reducing agent), and resolved by 3-12% Tris-acetate SDS-PAGE and analyzed by Western blotting.

Supplemental References

Anders S, Pyl PT, Huber W. 2015. HTSeq-A Python framework to work with high-throughput sequencing data. *Bioinformatics* **31**: 166–169.

Bray NL, Pimentel H, Melsted P, Pachter L. 2016. Near-optimal probabilistic RNA-seq quantification. *Nat Biotechnol* **34**: 525–527.

Dobin A, Davis CA, Schlesinger F, Drenkow J, Zaleski C, Jha S, Batut P, Chaisson M, Gingeras TR. 2013. STAR: Ultrafast universal RNA-seq aligner. *Bioinformatics* **29**: 15–21.

Durinck S, Moreau Y, Kasprzyk A, Davis S, De Moor B, Brazma A, Huber W. 2005. BioMart and Bioconductor: a powerful link between biological databases and microarray data analysis. *Bioinformatics* **21**: 3439–40.

Eden E, Navon R, Steinfeld I, Lipson D, Yakhini Z. 2009. GOrilla: A tool for discovery and visualization of enriched GO terms in ranked gene lists. *BMC Bioinformatics* **10**: 48 (2009) doi:10.1186/1471-2105-10–48.

Fazal FM, Han S, Parker KR, Kaewsapsak P, Xu J, Boettiger AN, Chang HY, Ting AY. 2019. Atlas of Subcellular RNA Localization Revealed by APEX-Seq. *Cell* **178**: 473-490.e26.

Gordon DE, Bond LM, Sahlender DA, Peden AA. 2010. A Targeted siRNA Screen to Identify SNAREs Required for Constitutive Secretion in Mammalian Cells. *Traffic* **11**: 1191–1204.

Huang DW, Sherman BT, Lempicki RA. 2009. Systematic and integrative analysis of large gene lists using DAVID bioinformatics resources. *Nat Protoc* **4**: 44–57.

Hug N, Cáceres JF. 2014. The RNA Helicase DHX34 Activates NMD by Promoting a Transition from the Surveillance to the Decay-Inducing Complex. *Cell Rep* **8**: 1845–1856.

Jagannathan S, Nwosu C, Nicchitta C V. 2011. Analyzing mRNA localization to the endoplasmic reticulum via cell fractionation. *Methods Mol Biol* **714**: 301–21.

Jan CH, Williams CC, Weissman JS. 2014. Principles of ER cotranslational translocation revealed by proximity-specific ribosome profiling. *Science* **346**: 1257521.

Love MI, Huber W, Anders S. 2014. Moderated estimation of fold change and dispersion for RNA-seq data with DESeq2. *Genome Biol* **15**: 550.

Piñol-Roma S, Dreyfuss G. 1992. Shuttling of pre-mRNA binding proteins between nucleus and cytoplasm. *Nature* **355**: 730–2.

Ran FA, Hsu PD, Wright J, Agarwala V, Scott DA, Zhang F. 2013. Genome engineering using the CRISPR-Cas9 system. *Nat Protoc* **8**: 2281–2308.

Reid DW, Nicchitta C V. 2012. Primary role for endoplasmic reticulum-bound ribosomes in cellular translation identified by ribosome profiling. *J Biol Chem* **287**: 5518–5527.

Schindelin J, Arganda-Carreras I, Frise E, Kaynig V, Longair M, Pietzsch T, Preibisch S, Rueden C, Saalfeld S, Schmid B, et al. 2012. Fiji: An open-source platform for biological-image analysis. *Nat Methods* **9**: 676–682.
